# Synovial tissue transcriptomes of long-standing rheumatoid arthritis are dominated by activated macrophages that reflect microbial stimulation

**DOI:** 10.1038/s41598-020-64431-4

**Published:** 2020-05-13

**Authors:** Biljana Smiljanovic, Andreas Grützkau, Till Sörensen, Joachim R. Grün, Thomas Vogl, Marc Bonin, Pascal Schendel, Bruno Stuhlmüller, Anne Claussnitzer, Sandra Hermann, Sarah Ohrndorf, Karlfried Aupperle, Marina Backhaus, Andreas Radbruch, Gerd R. Burmester, Thomas Häupl

**Affiliations:** 1 Department of Rheumatology and Clinical Immunology, Charité Universitätsmedizin, Berlin, Germany; 2 Deutsches Rheuma-Forschungszentrum Berlin (DRFZ), a Leibniz Institute, Berlin, Germany; 3 Institute of Immunology, Westfälische Wilhelms-Universität Münster, Münster, Germany

**Keywords:** Inflammation, Innate immunity, Rheumatology

## Abstract

Advances in microbiome research suggest involvement in chronic inflammatory diseases such as rheumatoid arthritis (RA). Searching for initial trigger(s) in RA, we compared transcriptome profiles of highly inflamed RA synovial tissue (RA-ST) and osteoarthritis (OA)-ST with 182 selected reference transcriptomes of defined cell types and their activation by exogenous (microbial) and endogenous inflammatory stimuli. Screening for dominant changes in RA-ST demonstrated activation of monocytes/macrophages with gene-patterns induced by bacterial and fungal triggers. Gene-patterns of activated B- or T-cells in RA-ST reflected a response to activated monocytes/macrophages rather than inducing their activation. In contrast, OA-ST was dominated by gene-patterns of non-activated macrophages and fibroblasts. The difference between RA and OA was more prominent in transcripts of secreted proteins and was confirmed by protein quantification in synovial fluid (SF) and serum. In total, 24 proteins of activated cells were confirmed in RA-SF compared to OA-SF and some like CXCL13, CCL18, S100A8/A9, sCD14, LBP reflected this increase even in RA serum. Consequently, pathogen-like response patterns in RA suggest that direct microbial influences exist. This challenges the current concept of autoimmunity and immunosuppressive treatment and advocates new diagnostic and therapeutic strategies that consider microbial persistence as important trigger(s) in the etiopathogenesis of RA.

## Introduction

Pathomechanisms, which lead to the ultimate outbreak of RA, remain unknown^1–3^. Nevertheless, early diagnosis and immunosuppressive treatment in rheumatoid arthritis (RA) are able to control inflammation and to reduce irreversible damage of the joints^4,5^. Rheumatoid factor (RF) and/or anti-citrullinated peptide antibodies (ACPA) may appear several years before the onset of clinical symptoms, suggesting that antigenic triggers precede the disease and can be successfully “controlled” by the immune system for a long time.

Microbial exposure and smoking are environmental factors, which in combination with genetic factors like HLA-DRB1 shared epitope and other risk alleles (e.g. PTPN22) play an important role in RA pathogenesis^6,7^. The search for pathogenic triggers in synovial fluid and tissue from RA patients has been inconclusive so far. Microbial DNA or antigen fragments were repeatedly reported in joints of RA and even of OA patients^8–10^. Furthermore, it was found that ACPA-positive RA patients react with citrullinated microbial epitopes, generated with microbial or human peptidylarginine deiminase^11^. Mucosal surfaces of gut, lung, and oral cavity have been implicated in RA pathogenesis since they provide a large surface for extensive interactions between commensal and pathogenic microbes from one side and host immune cells from the other^12^. So far, the epidemiological data demonstrated the relation between smoking, risk alleles, and ACPA positive RA. Microbiota studies associated gut dysbiosis with RA and revealed loss of microbial diversity when compared to healthy donors^13–17^. Besides considering bacterial pathogens like *Prevotella copri* or *Collinsella* genus as potential triggers, fungal β-glucan preparation zymosan A may also induce severe arthritis^14,17,18^. Thus, hypotheses of i) mucosal triggered antigen-specific immunity that cross-reacts with joint antigens (autoimmunity) or ii) expansion of pathogens on mucosal surfaces with release of immunostimulatory antigens and metabolites that are passing the mucosal barrier and spread into joints (permanent triggering) are discussed, which both may explain development towards chronic synovitis^8,12,19–22^.

**Figure 1:**
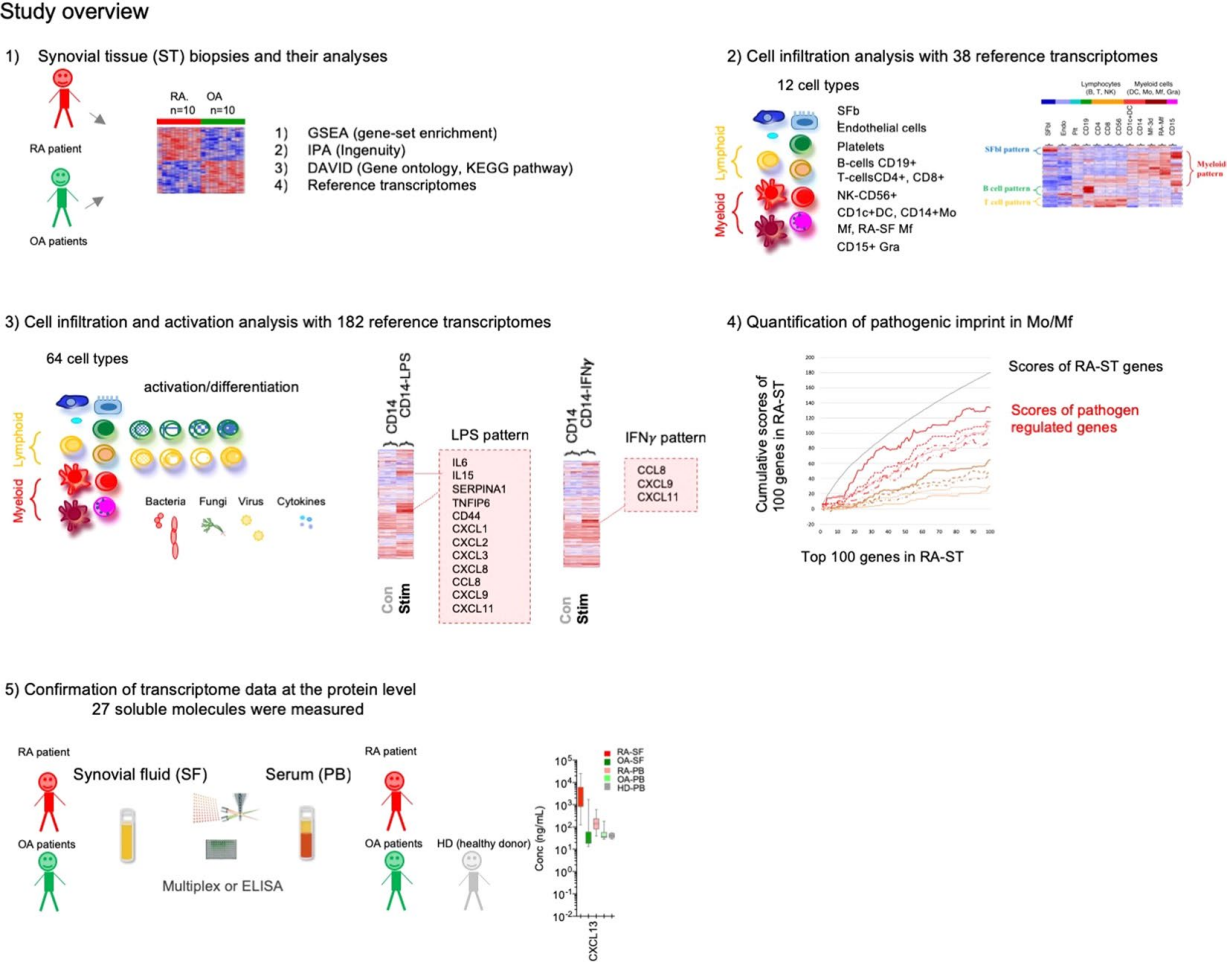
Overview of the study. (1) Synovial tissue (ST) biopsies from rheumatoid arthritis (RA) and osteoarthritis (OA) patients were profiled for gene expression with Affymetrix HG-U133A arrays. Pair-wise comparisons between 10 RA-ST and 10 OA-ST were performed by applying the BioRetis workflow, and the obtained transcriptome profiles were analyzed for differentially expressed genes with gene-set enrichment analysis (GSEA), Ingenuity pathway analysis (IPA), DAVID and reference transcriptomes. (2) Search for the gene-patterns of cells that infiltrate synovial tissues in RA-ST and OA-ST was performed with 38 reference transcriptomes of 12 cell types including: synovial fibroblasts (SFbl), endothelial cells (EC), platelets (Plt), B-, T-, NK-cells, monocytes, macrophages, DC and granulocytes. (3) This initial cell type screening with 38 transcriptomes was extended to stimulation and differentiation patterns with 182 reference transcriptomes that portrayed 64 different cell conditions including differentiation and activation of lymphoid cells as well as activation of myeloid cells with bacterial, fungal, viral pathogens and various inflammatory mediators (TNF, IL15, IL1β, IL4, IL10, IFNα, IFNγ). (4) Quantitative assessment of cell type specific and stimulus specific activation in RA-ST. (5) Validation of transcriptome data by selecting secreted molecules from RA-ST profile and determining these proteins in synovial fluid and serum from RA and OA patients.

In this study we aimed to characterize the immune response in the joints with respect to innate or adaptive immune dominance and to patterns of cell activation by defined cytokine or pathogen triggers (Fig. 1). Transcriptomes of highly inflamed synovial tissue (ST) samples from long-lasting RA were compared to osteoarthritis (OA). To relate transcriptional differences between RA and OA to immune cells and mechanisms of activation, 42 transcriptome data generated in our lab and relevant experiments collated from public GEO repositories were screened and a selected set of 182 reference transcriptomes was applied for pattern matching and quantification. This included resting, activated and differentiated cells of innate and adaptive immunity, synovial fibroblasts, endothelial cells and platelets. RA-ST specific transcripts mostly overlapped with monocyte/macrophage patterns that are activated by bacterial and fungal pathogens or their components (LPS, zymosan) and that are amplified but only partially induced by inflammatory mediators like TNF, IFNγ, IL1β, IL15 or alarmin S100A8. Patterns of infiltrated lymphocytes were evident only in RA-ST. In contrast, OA-ST specific transcripts overlapped with patterns of differentiating macrophages and fibroblasts. These changes were confirmed by detecting the corresponding inflammation related proteins in synovial fluid of RA but not OA patients. Although these proteins were diluted and in part neutralised in the blood, these differences between RA and OA were even evident in serum.

## Results

### RA-ST transcriptomes indicate involvement of both innate and adaptive immunity

**Figure 2:**
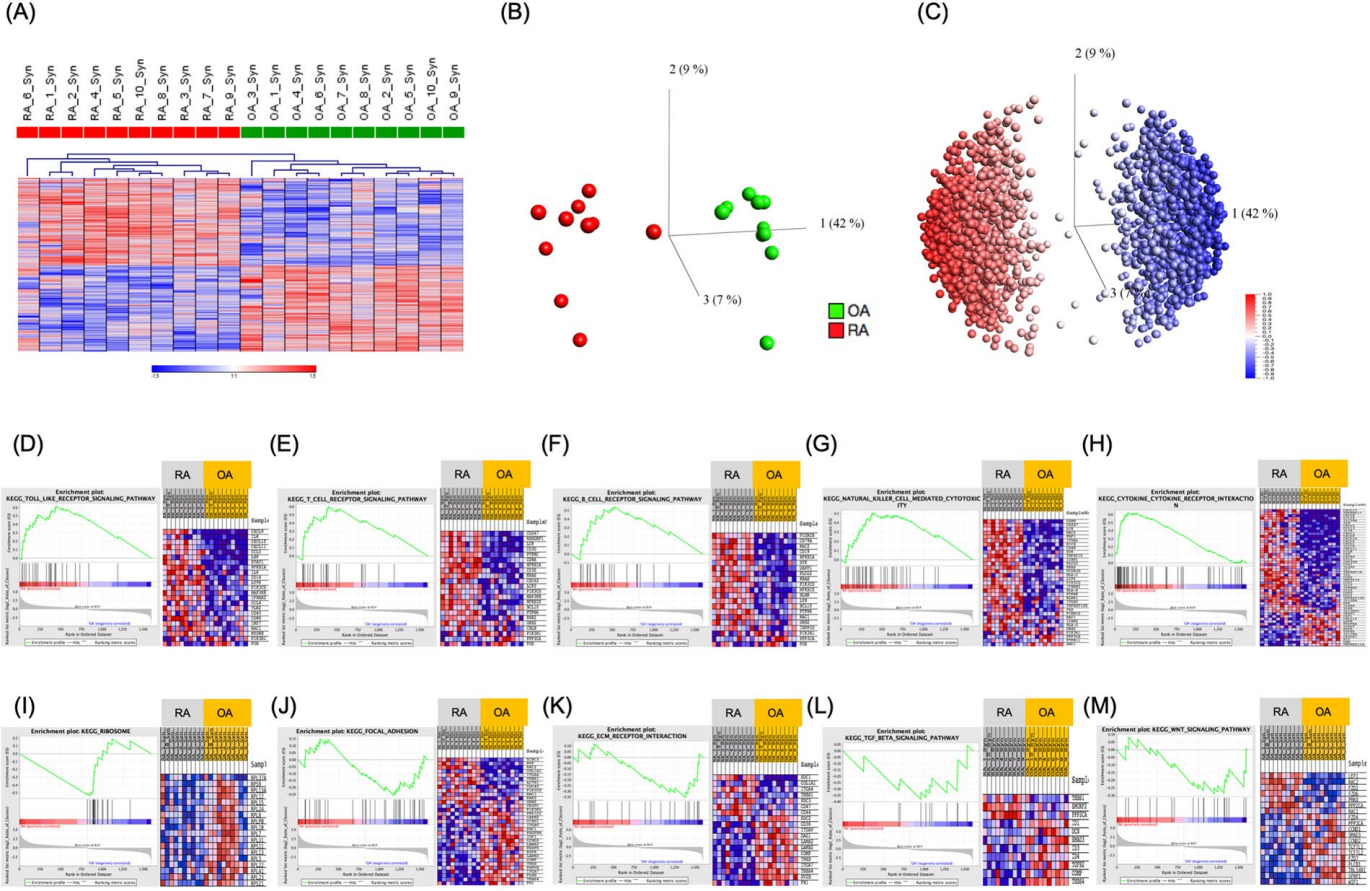
Transcriptome profiles distinguish RA- from OA-synovial tissues (ST) and reveal involvement of innate and adaptive immunity in RA pathogenesis. (**A**) Hierarchical clustering (Euclidean distance, average linkage) with 2019 differentially expressed probe-sets (rows), which were identified by pair-wise comparisons between RA (n = 10) and OA (n = 10) synovial tissue transcriptomes, separate RA from OA samples (columns). Signals were log-transformed and z-normalized for each probe set to display relative intensities as indicated by the scale bar. Principal component analysis (PCA) was performed for the 20 samples based on the differentially expressed genes (**B**). Based on this PCA, a synchronized representation of the 2019 probe-sets is displayed in (**C**). The first 3 principal components, PC1, PC2, and PC3, reflect 42%, 9% and 7% of variance, respectively. Samples from RA patients were coloured in red, those from OA in green (**A** and **B**). Probe-sets highest in RA-ST (n = 1010) are red and those highest in OA-ST (n = 1009) are blue (**A** and **C**). Gene set enrichment analysis (GSEA) of the 2019 differentially expressed probe-sets identified particular KEGG pathways for RA-ST and OA-ST, which suggest different pathomechanisms in these two diseases. The KEGG pathways presented here with enrichment plots and heatmaps of gene-sets accentuated the role of innate immunity, cytokines, B-, T- and NK-cells in RA pathogenesis (**D**–**H**) and tissue damage without substantial activation of the immune system in OA (**I**–**M**).

Samples of highly inflamed synovial tissue (ST) areas from RA and representative specimens from OA patients were collected during open surgery. Transcriptome comparisons identified extensive differences in RNA expression. 2019 Affymetrix probe-sets (~1580 genes) were selected, 1010 up- and 1009 down-regulated (supplementary table 1). Hierarchical clustering (HC) and principal component analysis (PCA) of these transcripts demonstrated a clear separation between these two diseases (Fig. 2A–C). Specificity of RA-ST genes when compared to OA-ST, which is frequently considered as control group because of limited accessibility to normal synovial tissue, was confirmed when RA-ST was compared to synovial tissues samples obtained i) after joint trauma or ii) post mortem from tissue donors. However, both types of “normal” synovial tissues displayed some abnormalities, like gene-patterns of unspecific inflammation or hypoxia induced alterations (supplementary figure 1).

Functional annotation of 2019 transcripts with GSEA, IPA and DAVID suggested infiltration and activation of immune cells, which differs between RA- and OA-ST and is well known from histological investigations^23,24^. In RA-ST, GSEA annotated expression of transcripts to TLR-, T-, B-, and NK-signalling pathways and emphasised involvement of various cytokines (supplementary table 2; Fig. 2D–H). In OA-ST, GSEA suggested alterations in processes related to focal adhesion and extracellular matrix organisation and involvement of TGFβ- and WNT-signalling pathways (supplementary table 2; Fig. 2I–M). Correspondingly, IPA pointed in RA-ST to TCR-, complement-, TNF-, IFN-, and IL6-receptor mediated signalling as well as chemotactic and inflammatory processes related to CXCL9, CXCL10, CXCL11, CXCL13, IL15, IL32, and CCL13 (supplementary figures 2-3).

### Cellular deconvolution of RA-ST and OA-ST transcriptomes using cell type specific reference transcriptomes

**Figure 3:**
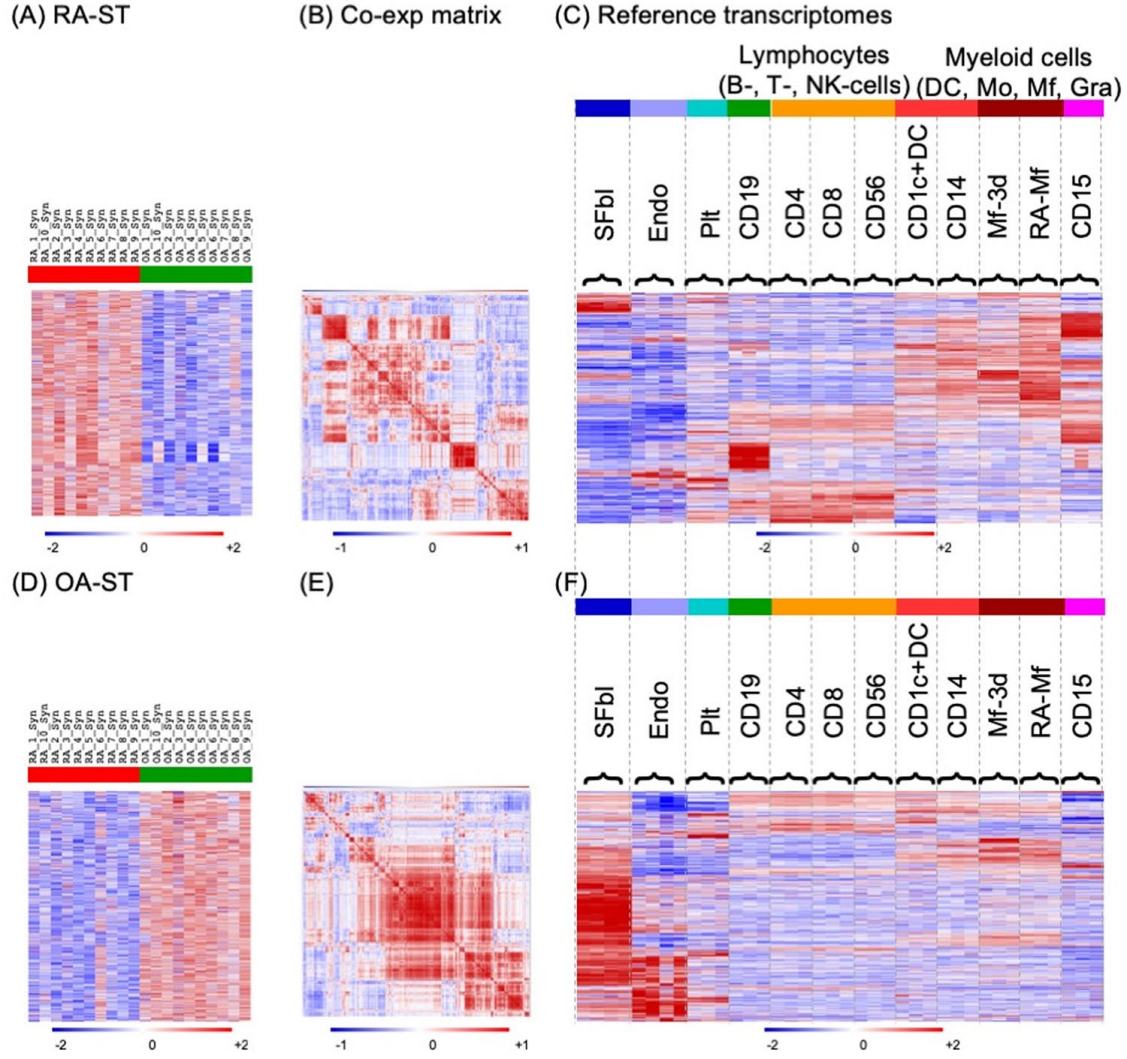
Searching for cell type specific gene-patterns identified a dominance of monocyte/macrophage infiltration in RA-ST and of synovial fibroblasts in OA-ST. In total, 38 reference transcriptomes (**C** and **F**) were applied for analysis of up-regulated probe-sets in RA-ST (n = 1010) **(A)** and OA-ST (n = 1009) (**D**). These included: synovial fibroblasts (SFbl) (n = 4, 2 from RA and 2 from OA patients; dark blue), endothelial cells (EC) (n = 4; light blue), platelets (Plt) (n = 3; cyan), CD19^+^B cells (n = 3; green), CD4^+^T cells (n = 3; yellow), CD8^+^T cells (n = 3; yellow), CD56^+^NK cells (n = 3; yellow), CD1^+^DC (n = 3; red), CD14^+^ monocytes (n = 3; red), macrophages (n = 3; differentiated for 3 days from blood monocytes of healthy donors; dark red), macrophages isolated from synovial fluid of RA patients (n = 3; dark red) and CD15^+^ granulocytes (n = 3; pink). The overview of reference transcriptomes is provided in supplementary table 4. Co-expression matrices (**B**) and (**E**) were generated by correlating expression of the 1010 and 1009 probe-sets in the reference transcriptomes **C** and **F**, respectively. These matrices of correlation coefficients were hierarchically clustered to group co-expressed genes for pattern search in the reference transcriptomes. This order of genes was applied to sort probe-sets in RA-ST and OA-ST (**A** and **D**) and in reference transcriptomes (**C** and **F**). This alignment identified the patterns, which were characteristic for different cell types.

For a more detailed analysis, transcripts specific for RA-ST (Fig. 3A) and OA-ST (Fig. 3D) were compared to expression patterns in reference transcriptomes of defined cell types that could be inferred from GSEA and DAVID annotations (supplementary tables 2 and 3). In total, 38 reference transcriptomes of 12 cell types were selected from own resources and from the GEO database, including synovial fibroblasts, endothelial cells, platelets, CD19^+^B-, CD4^+^T-, CD8^+^T-, CD56^+^NK-cells, CD1c^+^DC, CD14^+^ monocytes (Mo), macrophages (Mf), Mf from RA synovial fluid (RA-SF) and CD15^+^ granulocytes (supplementary table 4)^25–31^. Expression levels of the RA-ST and OA-ST specific transcripts were selected from the different reference transcriptomes to calculate their co-expression in these reference transcriptomes by Pearson correlation (matrices 3B and 3E). Sorting the genes according to the cluster of the correlation coefficient matrix identified cell type specific gene-patterns, which differed between RA-ST and OA-ST. RA-ST was dominated by expression patterns of myeloid cells including Mf from RA-SF, *in vitro* differentiated Mf, blood monocytes and granulocytes, while OA-ST revealed an extensive gene-pattern of synovial fibroblasts and minor ones of endothelial cells and *in vitro* differentiated Mf (Fig. 3C,F). Typical B- and T-lymphocyte gene-patterns with immunoglobulin and TCR genes, respectively appeared only in RA-ST. Two leukocyte gene-patterns appeared in OA-ST: 1) common for monocytes, CD1c^+^DC and lymphocyte and 2) characteristic for monocytes and Mf. Thus, infiltration of monocytes/Mf, both in RA-ST and OA-ST, and lymphocytes only in RA-ST indicates significant differences in the involvement of the immune system in these two diseases”.

### RA-ST transcriptomes show activation patterns that indicate macrophage activation by microbial triggers and inflammatory mediators

**Figure 4:**
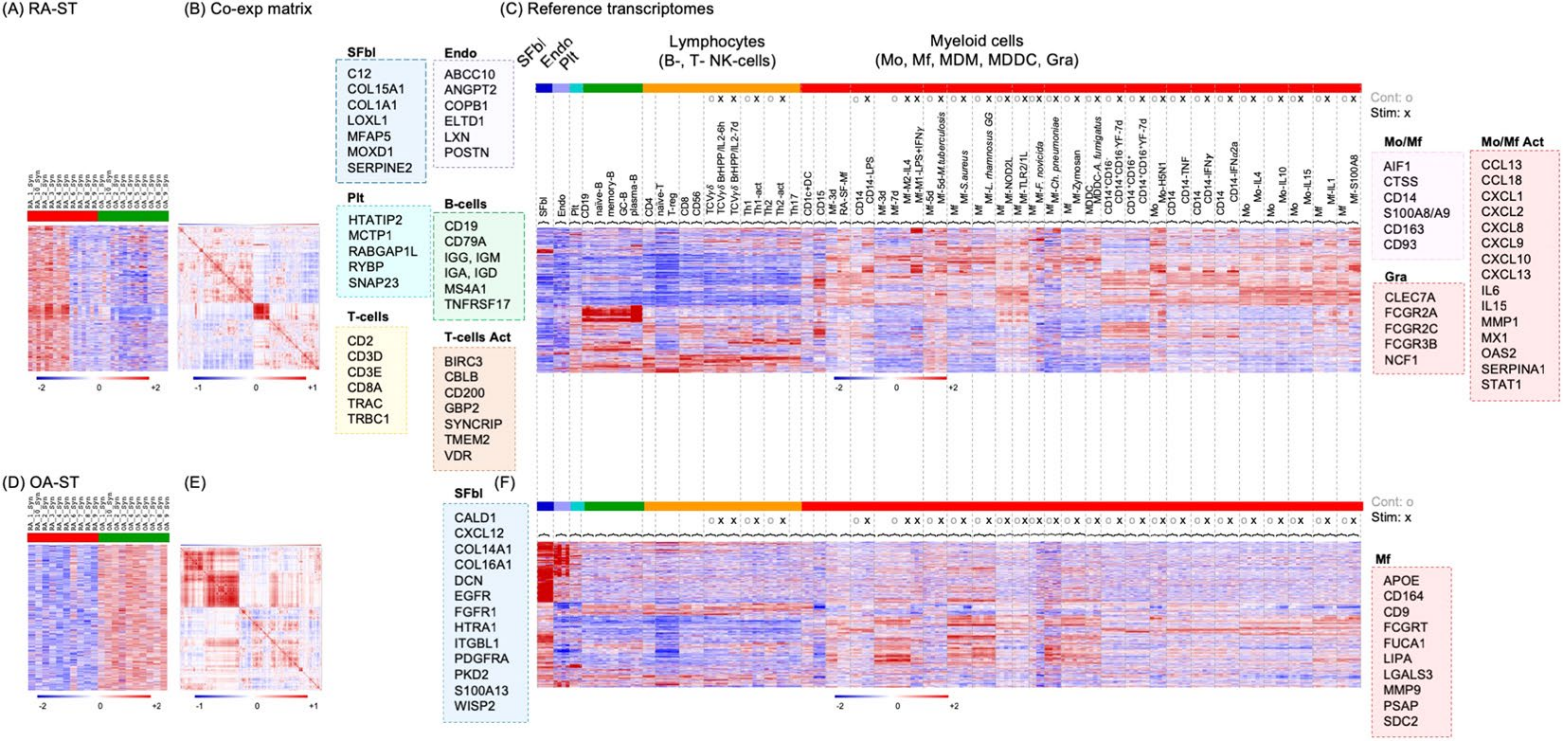
Co-expression analyses with reference transcriptomes of activated cell types identified monocyte/macrophage responses in RA-ST that overlapped with those triggered by microbes. Up-regulated probe-sets (n = 1010) in RA-ST (n = 10) **(A)** and up-regulated probe-sets (n = 1009) in OA-ST (n = 10) (**D**) were analysed with 203 reference transcriptomes (182 different reference transcriptomes with repeated control samples for different stimuli of the same experiment; listed in supplementary tables 4 and 5). In brief, the previous 38 reference transcriptomes used to define the main populations of immune cells (Fig. 3), were extended by additional populations of B-cells, T-cells, and monocytes (Mo), Mf, monocyte derived Mf (MDM) and monocyte derived DC (MDDC), which were activated with different stimuli. These were applied to calculate and cluster the co-expression matrices (**B** and **E**) and to align patterns with expression in RA-ST and OA-ST (**A** and **D**) and with the reference transcriptomes (**C** and **F**). **Colour codes:** Synovial fibroblasts (SFbl) (n = 4, dark blue), Endothelial cells (EC) (n = 4, light blue), Platelets (Plt) (n = 3, cyan); B cell types (green): CD19^+^(n = 3), naïve- (n = 3), memory- (n = 3), germinal centre-B cells (n = 3) and plasma cells (n = 3); T cell types (yellow): CD4^+^ (n = 3), CD8^+^ (n = 3), naïve- (n = 3), regulatory- (n = 3), resting (n = 9) and activated: γδ- (n = 3), Th1- (n = 3), Th2- (n = 3), and Th17-cells (n = 3). Activation of γδT was performed with the phosphoantigen BrHPP and IL2, while Th1 and Th2 cells were activated with PMA/Ionomycin; CD56^+^NK cells (n = 3, yellow); Myeloid cells (red): Mo, Mf, MDM, MDDC, CD1^+^DC and CD15^+^ granulocytes and their activation by broad spectrum of stimuli including: **(1) Bacterial stimuli:** (1) *Mycobacterium tuberculosis*, (2) *Staphylococcus aureus*, (3) *Lactobacillus rhamnosus* LGG, (4) NOD2L stimulation (muramyl dipeptide), (5) TLR2/1 L stimulation (triacylated lipopeptide), (6) *Francisella novicida*, and (7) *Chlamydia pneumoniae*. **(2) Fungal stimuli:** (1) zymosan A and (2) *Aspergillus fumigatus*. **(3) Viral stimuli:** (1) live attenuated viral strain of yellow fever vaccine and (2) H5N1 influenza. **(4) Cytokines:** TNF, LPS, IFNγ, IFNα2a, IL4, IL10, IL15 and IL1β. **(5) Alarmin:** S100A8. **(6)** ***In vitro*** **polarisation:** of monocytes to M1-Mf or M2-Mf were performed with LPS + IFNγ and IL4, respectively.

To investigate immune activation of various types of leukocytes in RA-ST and OA-ST, the initial analysis with the cell type specific transcriptomes was extended to 182 reference transcriptomes (supplementary table 4). These included i) B cells (naïve-, memory-, germinal centre B-cells and plasma cells), ii) T-cells (Th1, Th2, Th17, naïve T-cells, regulatory T-cell and γδT-cells), and iii) myeloid cells stimulated for differentiation or activation by various microbial and inflammatory stimuli. For each stimulation experiment an unstimulated control was included, which allowed identification of overlapping and stimulus-specific gene-patterns (Fig. 4C,F and supplementary table 5).

The most obvious differences between RA-ST (Fig. 4A) and OA-ST (Fig. 4D) were gene-patterns of typical innate immune cell activation in RA-ST (Fig. 4C), and patterns of differentiated but not activated Mf in OA-ST (Fig. 4F). In RA-ST, myeloid activation patterns overlapped with response pattern induced by bacterial pathogens or components like LPS, M1-Mf, *M. tuberculosis*, *S. aureus*, *L. rhamnosus*, NOD2-ligand (muramyl dipeptide), TLR2/1-ligand (triacylated lipopeptide), *F. tularensis* subsp. *novicida* or *C. pneumoniae*. Activation patterns triggered *in vitro* in Mf by the fungus *A. fumigatus* or the fungal component zymosan A appeared to be weaker by intensity and number of transcripts. Activation patterns triggered in monocytes by viral stimuli including influenza virus (H5N1) *in vitro* and yellow fewer vaccine (YFV) *in vivo* showed only a weak overlap with RA-ST profiles. Although H5N1 activation of monocytes showed patterns that overlapped with those triggered by some bacterial stimuli, it should be noted that the profile of control monocytes in this experiment exhibited increased patterns when compared to control samples from other experiments, suggesting monocyte activation already in cell culture or during isolation. In general, H5N1 and YFV stimulation revealed a minor set of genes that was part of the IFN induced response (IFNα or IFNγ). The gene-patterns associated with IFNγ and IFNα stimulation of monocytes were small by the number of genes but strong with respect to their expression. TNF, IL15 and IL1β related monocyte stimulation patterns were similar but weaker in intensity and smaller in number of genes when compared with bacterial stimuli. RA-ST did not reveal substantial patterns related to IL4 and IL10 stimulation of monocytes.

A pattern with dominance in granulocytes was evident only in RA-ST and included molecules involved in phagocytosis, complement activation, pathogen recognition, alarmins, and cytosolic factors (Fig. 4C). Although dominant in granulocytes, this pattern overlapped with Mf from RA-SF, blood monocytes and *in vitro* differentiated Mf.

The gene-pattern related to B-cells in RA-ST in the previous analysis, was common for CD19+, naïve-, memory-, germinal centre B-cells and plasma cells but exhibited the highest expression in plasma cells (Fig. 4C).

The T-cell gene-patterns of RA-ST suggested infiltration of naïve, T-reg, activated and differentiated T-cells (Fig. 4C). The gene-pattern of activated T-helper subsets (Th1, Th2 and Th17) reflected a general activation by TCR signalling, because all these T-cell reference transcriptomes were experimentally triggered by PMA/Ionomycin^32^.

In OA-ST, the most prominent gene-patterns were related to fibroblasts and differentiated myeloid cells, more precisely monocyte derived macrophages (MDM) and monocyte derived DC (MDDC) (Fig. 4F). This myeloid pattern partially overlapped with expression patterns of synovial fibroblasts, and did not exhibit activation patterns related to pathogens or inflammatory mediators. B- or T-cell specific transcripts were not identified in OA-ST.

### Monocyte response to microbes is quantitatively the leading functional response in RA-ST

**Figure 5:**
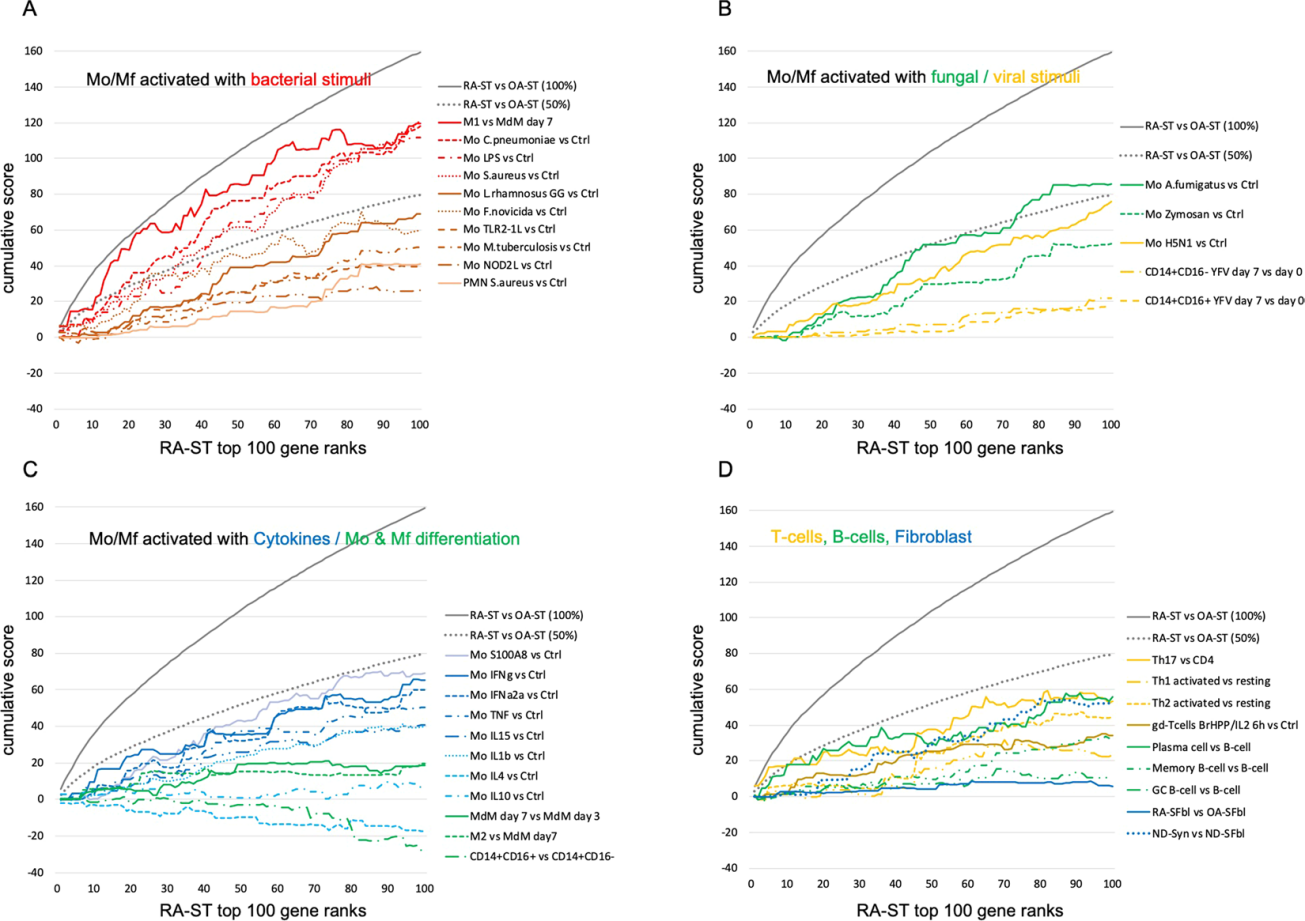
Quantitative assessments of cell-type and stimulus specific activation of the top 100 genes in RA-ST. Out of the 1010 probe-sets up-regulated in RA-ST vs OA-ST, the top 100 genes with the highest score values (determined by SLR and frequency of changes for each gene) were selected and ranked by scores. These ranks are represented on the abscissa. The ordinate shows the cumulative sum of scores as it changes with each additional rank and its gene. As target value, the grey line indicates the cumulative sum of scores of RA-ST (100% score) and as auxiliary value, the dotted grey line indicates its 50% value. For the genes on the abscissa, we applied the scores obtained from different comparisons between reference transcriptomes as indicated in the legend to the right. (**A**) presents the cumulative sum of scores for bacterial activation of monocytes/macrophages with LPS and IFNγ (M1-Mf), *C. pneumoniae*, LPS, *S. aureus*, *L. rhamnosus*, *F. novicida*, TLR1/2 L, *M. tuberculosis*, NOD2L and granulocytes with *S. aureus*. (**B**) indicates the scoring achieved by fungal and viral activation in monocytes/macrophages induced by *A. fumigatus*, zymosan A, H5N1 influenza virus and yellow fever vaccination in classical (CD14^++^CD16^−^) and non-classical (CD14^+^CD16^+^) monocytes. (**C**) shows the scoring from reference comparisons (supplementary table 6) that present cytokine induced activation and differentiation in monocytes/macrophages. It included profiles of TNF, IFNγ, IFNα2a, IL4, IL10, IL15, IL1β and S100A8 stimulation, of macrophage differentiation with CSF2 for 7 days and of non-classical (CD14^++^CD16^−^) compared to classical (CD14^++^CD16^−^) blood monocytes. (**D**) outlines the scoring for activated T-cells, B-cells and synovial fibroblasts (SFbl). Activation of T-cells was determined in comparisons between Th1 activated vs Th1 resting, Th2 activated vs Th2 resting, and Th17 activated vs CD4 T-cells. B-cell profiles were determined in comparisons between plasma-, memory B- and germinal center (GC-) B-cells to naïve B-cells. Profiles of synovial fibroblast were determined in comparisons of *in vitro* cultured RA-SFbl and OA-SFbl and of native synovial tissue compared with *in vitro* cultured SFbl, both from normal joints of tissue donors (collected early post mortem).

To quantify the different stimulation patterns in RA-ST transcriptomes, differences between RA-ST and OA-ST, and between reference stimulation experiments and their controls (35 reference comparisons; supplementary table 6) were calculated and scored for each probe-set in each comparison as described in the supplementary file. The 1010 RA-ST specific probe-sets were selected, reduced to one representative probe-set per gene, and ranked by scores determined in RA-ST vs OA-ST comparisons. For this RA-ST defined order of genes, cumulative scores were calculated for each reference comparison and displayed for the top 100 genes (Fig. 5A–D). This revealed that the top 100 RA-ST genes were best represented by those induced in M1 macrophages (generated by LPS and IFNγ stimulation) with scores up to 96% of RA-ST score, and in monocytes stimulated with *S. aureus*, *C. pneumoniae*, LPS or *A. fumigatus*, which reached up to 76%, 76%, 73% and 59% of RA-ST scores, respectively (supplementary table 7, worksheet-A). All other conditions of monocyte stimulation and differentiation as well as lymphocyte or granulocyte related reference comparisons did not exceed 50% of RA-ST scores. Similar scores and rankings were calculated for OA-ST probe-sets and demonstrated that none of these inflammatory reference stimulation conditions were detectable in OA-ST (supplementary figure 4A–D). Instead, the top 100 OA-ST genes (supplementary table 7, worksheet-C) revealed association with non-inflammatory macrophage differentiation (MDM) and were best represented by the normal synovial tissue transcriptome when compared to the condition of cultured and proliferating synovial fibroblasts. This corresponds to a rather normal tissue phenotype in OA when compared to RA, where normal synovium related patterns were significantly underrepresented (Fig. 5D).

To estimate whether the RA-ST genes have a significant influence in the 35 reference comparisons (supplementary table 6), we applied the RA-ST scores to the top 100 genes of each reference comparison. Again, the cumulative RA-ST scores calculated for the top genes of M1-macrophage phenotype and monocytes stimulated with *C. pneumoniae* or *S. aureus* reached highest values and reached up to 1/3 of the expected value given by the score between RA-ST and OA-ST (supplementary figure 5A–D). This indicates that many of the leading candidate genes in RA-ST overlap with those in microbial induced reference comparisons. In fact, out of the top 100 genes selected for each comparison (supplementary table 7, worksheet-E), 20 RA-ST genes overlapped with M1-macrophages, 15 with monocytes after stimulation with *L. rhamnosus*, 22 with *S. aureus*, 20 with *C. pneumon*iae and also 10 with zymosan A.

### RA-ST transcripts of secreted proteins are predominantly increased in monocytes/macrophages stimulated with microbial components

Besides cell-cell interactions via surface molecules, secreted mediators are crucial for the inflammatory response. Thus, focussing on transcripts of secreted proteins that are annotated by gene ontology as “extracellular space” and “extracellular exosome” (GO:0005615 and GO:0070062; supplementary table 3) we were aiming to identify stimulation induced gene-patterns in RA-ST that suggest secreted markers for further confirmatory investigations at protein level.

**Figure 6:**
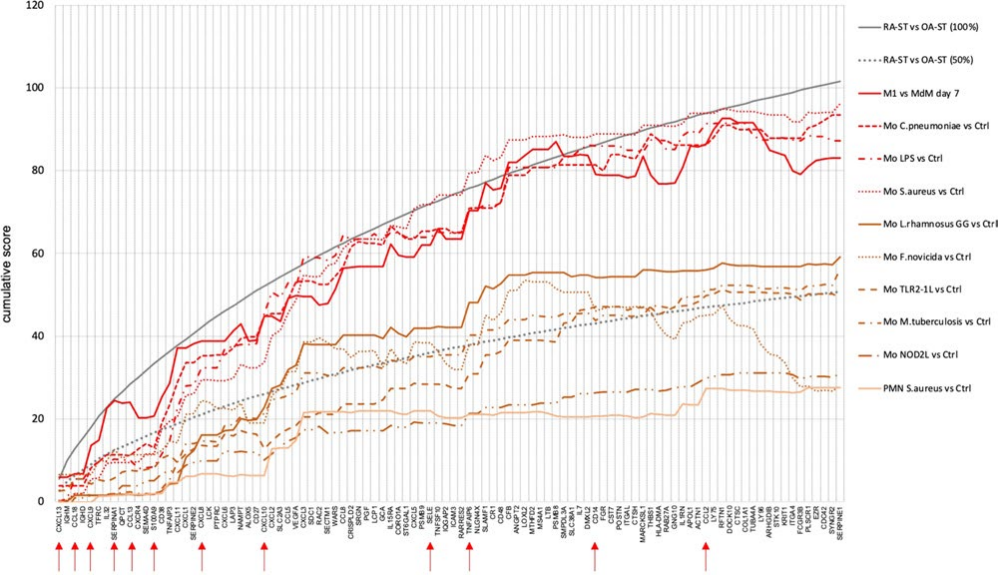
Quantitative assessment of bacteria induced activation in the top 100 genes encoding secreted/shedded molecules in RA-ST. In total, 345 out of the 1010 probe-sets up-regulated in RA-ST encoded secreted/shedded molecules. Out of these 345 probe-sets, the top 100 genes with the highest score values were selected and ranked by scores. These ranks with gene names are represented on the abscissa. The ordinate shows the cumulative sum of scores as it changes with each newly added gene. As target value, the grey line indicates the cumulative sum of scores for secreted/shedded molecules of RA-ST (100% score) and as auxiliary value, the dotted grey line indicates its 50% value. We applied the scores obtained from different comparisons between stimulated reference transcriptomes and unstimulated controls as indicated in the legend to the right: monocytes/macrophages stimulated with LPS and IFNγ (M1-Mf), *C. pneumoniae*, LPS, *S. aureus*, *L. rhamnosus*, *F. novicida*, TLR1/2L, *M. tuberculosis*, NOD2L and granulocytes with *S. aureus*. Red arrows indicate selected transcripts of secreted molecules, which were validated at the protein level.

About 1/3 of the transcripts belonged to these GO annotations, 345 of the 1010 probe-sets (256 genes) were increased in RA-ST (supplementary figures 6A, 6G) and 405 of the 1009 probe-sets (244 genes) were increased in OA-ST (supplementary figures 6D and 6J). Clustering these genes in the reference transcriptomes largely resembled the distribution of gene-patterns identified for all 2019 probe-sets differentially expressed between RA-ST and OA-ST (Figs. 3–4). Myeloid cells with activation patterns triggered by bacterial/fungal pathogens or inflammatory mediators dominated in RA-ST, while differentiated macrophages and fibroblasts were typical for OA-ST (supplementary figures 6G–L). Comparative quantification of the top 100 secreted proteins of RA-ST in the different reference transcriptomes by scores revealed again type M1-macrophage, *S. aureus*, *C. pneumoniae* and LPS stimulation of monocytes as the leading conditions in RA-ST (Fig. 6), which even exceeded in part the cumulative score of the 100 top genes from the whole RA-ST transcriptome (Fig. 5). *A. fumigatus* induced scores in monocytes were weaker but still reached up to 82% of RA-ST scores for secreted proteins. Th17 differentiation and monocyte stimulation with IFNγ, H5N1 influenza virus, zymosan A or IFNα reached up to 73%, 55%, 54%, 53% or 50% of the RA-ST scores, respectively (supplementary figure 7 and supplementary table 7, worksheet-B). This demonstrates once again that stimulation of myeloid cells with microbes or their components mimics best the transcriptional profile of secreted proteins in RA-ST. Transcripts of secreted proteins of OA-ST again did not reveal any overlap with patterns of immune cell stimulation but corresponded best to the normal synovial tissue phenotype when compared to cultured synovial fibroblast (supplementary figure 8, supplementary table 7, worksheet-D), which again was underrepresented in RA-ST and may indicate loss of fibroblast differentiation in RA.

To investigate, which of the 35 reference comparisons have similar response patterns, we correlated them on the basis of their scores for the top 100 RA gene. Monocyte activation by bacterial, fungal, TNF, IL15 and IL1β stimulations clustered together (supplementary figure 9A). The cluster of viral and IFN stimulation was partially shared with M1-Mf and *C. pneumoniae* induced monocytes activation, which is expected for M1-Mf as these *in vitro* polarized cells were also activated with IFNγ in addition to LPS. T-cell patterns were also in part overlapping but formed their own cluster.

To clarify the impact of endogenous triggers on monocyte activation patterns similar to exogenous (bacterial/fungal) stimulation, reference transcriptomes were screened for expression of those cytokines that were used for monocyte stimulation (TNF, IL1β, IL4, IL10, IL15, IFNα, IFNγ, CSFs). It appeared that TNF, IL15 and IL1β, which were the cytokines that induced patterns similar to bacterial and fungal triggering, were also part of the innate response to bacterial and fungal stimulation of myeloid cells. Besides monocytes, TNF was also induced in activated Th1, Th2, and Th17 cells (supplementary figure 9B). However, these T-cells were stimulated by CD14^+^ monocytes as antigen presenting cells (Th17) or by PMA and ionomycin as a substitute (Th1, Th2)^32,33^. This suggested again that innate triggering of monocytes appears to be indispensable.

### Secreted proteins of activated myeloid cells in RA-ST are evident in RA synovial fluid and reflect RA synovitis in the blood

A selection of secreted proteins identified by this transcriptome analysis in RA-ST was measured in paired samples of synovial fluid (SF) and serum from RA and OA patients to validate the results of previous transcriptome analysis. These included cytokines (CCL2, CCL13, CCL18, CXCL9, CXCL10, CXCL13, IL8, TNFAIP6), alarmins S100A8/A9, enzymes (MMP3, A1AT), and molecules shedded from the cell surface (sCD14, sCD163, sCD44, PLAUR, sICAM1, sVCAM1, sSELE, sSELP). While the majority of selected molecules originated from activated myeloid cells in RA-ST (12 were within the top 100 of RA-ST transcripts coding secreted proteins in Fig. 6), we also included molecules that reflect activation of synovial fibroblasts (MMP3), endothelial cells (sSELE, sVCAM1), platelets (sSELP), activation of Th1- and Th17-cells (TNF, IFN, MIF), as well as early-differentiation of macrophages (SPP1). Additionally, S100P and IL1R2 were included as part of the gene-patterns from RA blood monocytes in our previous study^34^. In total, 27 proteins were determined in paired samples of SF and serum from 18 RA and 15 OA patients and in serum from 14 healthy donors (Table 1).

**Table 1:** Concentrations of 27 markers were measured in SF from RA (n = 18) and OA (n = 15) patients and in serum from RA (n = 18), OA (n = 15) and healthy donors (HD; n = 14).

Molecules	p values (RA-SF vs OA-SF)	p values (RA vs OA vs HD)	RA vs OA	RA vs HD	OA vs HD
sCD14	<0.0001 (****)	0.0005 (***)	***	*	ns
S100A8/9	<0.0001 (****)	0.0006 (***)	***	*	ns
S100P	<0.0001 (****)	0.0037 (**)	*	*	ns
LBP	<0.0001 (****)	0.0003 (***)	*	***	ns
CXCL13	<0.0001 (****)	<0.0001 (****)	***	****	ns
MMP3	<0.0001 (****)	<0.0001 (****)	****	***	ns
CCL18	0.004 (**)	0.0014 (**)	*	**	ns
sCD163	<0.0001 (****)	0.0267 (*)	ns	*	ns
uPAR	0.0041 (**)	0.0361 (*)	ns	*	ns
CCL2 (MCP1)	0.7685 (ns)	0.0009 (***)	ns	**	**
CXCL9 (MIG)	<0.0001 (****)	<0.0001 (****)	ns	****	*
CXCL10 (IP10)	<0.0001 (****)	0.0002 (***)	ns	***	*
MIF	0.0004 (***)	0.0001 (***)	ns	***	***
CCL13 (MCP4)	0.03 (*)	0.0292 (*)	ns	ns	*
A1AT	0.0131 (*)	0.0233 (*)	*	ns	ns
OPN (SPP1)	0.0052 (**)	0.0412 (*)	*	ns	ns
sVCAM1	0.0074 (**)	0.0177 (*)	*	ns	ns
sCD44	0.0002 (***)	0.1577 (ns)	ns	ns	ns
sICAM1	<0.0001 (****)	0.062 (ns)	ns	ns	ns
sE Selectin	0.0016 (**)	0.3804 (ns)	ns	ns	ns
sP Selectin	0.0021 (**)	0.304 (ns)	ns	ns	ns
IL1R2	0.0006 (***)	0.8727 (ns)	ns	ns	ns
TNFAIP6	0.0295 (*)	0.1587 (ns)	ns	ns	ns
IL8	<0.0001 (****)	##			
TNF	0.0473 (*)	##			
IFNγ	#	##			
IFNα	#	##			

Groups were compared by applying Mann-Whitney U-test for calculating p-values in comparisons between RA and OA in SF and Kruskal-Wallis test (with Dunn’s multiple comparisons test) for calculating p-values in comparisons between RA, OA and HD in sera. *p < 0.05; **p < 0.01; ***p < 0.001; ****p < 0.0001; ns: non-significant. ^#^Below detection limit in most of OA patients. ^##^Below detection limit in most of HD, and OA patients.

Mann-Whitney and Kruskal-Wallis (with Dunn's multiple comparisons test) were applied for calculating p-values in comparisons between RA and OA in SF and between RA, OA and HD in sera, respectively.

p-value less than 0.05 was considered as significant; * < 0.05; ** < 0.01; *** <0.001; **** < 0.0001; ns-non-significant.

^#^In most of OA patients values were below limit of detection; ## in most of HD and OA patients values were below limit of detection. RA-rheumatoid arthritis; OA-osteoarthritis, HD-healthy donors.

Out of these proteins, 23 were elevated in RA-SF when compared to OA-SF and confirmed the differential expression of transcripts. Only MCP4 (CCL13) was lower in RA-SF than in OA-SF and contrasted transcriptome data. Based on the concentrations of the 23 molecules and MCP4, the majority of RA patients were separated from OA patients by HC and PCA (supplementary figures 10A and 10C).

Compared to SF, serum levels of the 27 proteins were lower for the majority of the markers, indicating that most of the factors are produced at the site of inflammation in the arthritic joints, the place, from which they are distributed and appear diluted in the blood. Dilution and “neutralization” by binding to serum proteins, metabolism in the liver or excretion into urine reduce discriminatory power of these proteins in serum. Few molecules revealed higher concentrations in serum than SF (A1AT, IL1R2, LBP, sICAM1, sSELE, sSELP) suggesting other sites of their production than synovium, like liver for acute phase response proteins or endothelial cells for adhesion molecules. Both PCA and HC revealed incomplete separation by serum molecules between RA, OA and HD, and demonstrated the heterogeneity between RA patients. However, it was obvious that RA exhibited the greatest distance from HD, while OA patients were closer to HD than to RA. This confirmed the inflammation patterns in RA sera that match with pathogen-like stimulation of myeloid cells in RA-ST (supplementary figures 10B and 10D).

### Concentration of top proteins, which arise in RA joints and spread into blood, correlated with disease activity

**Figure 7:**
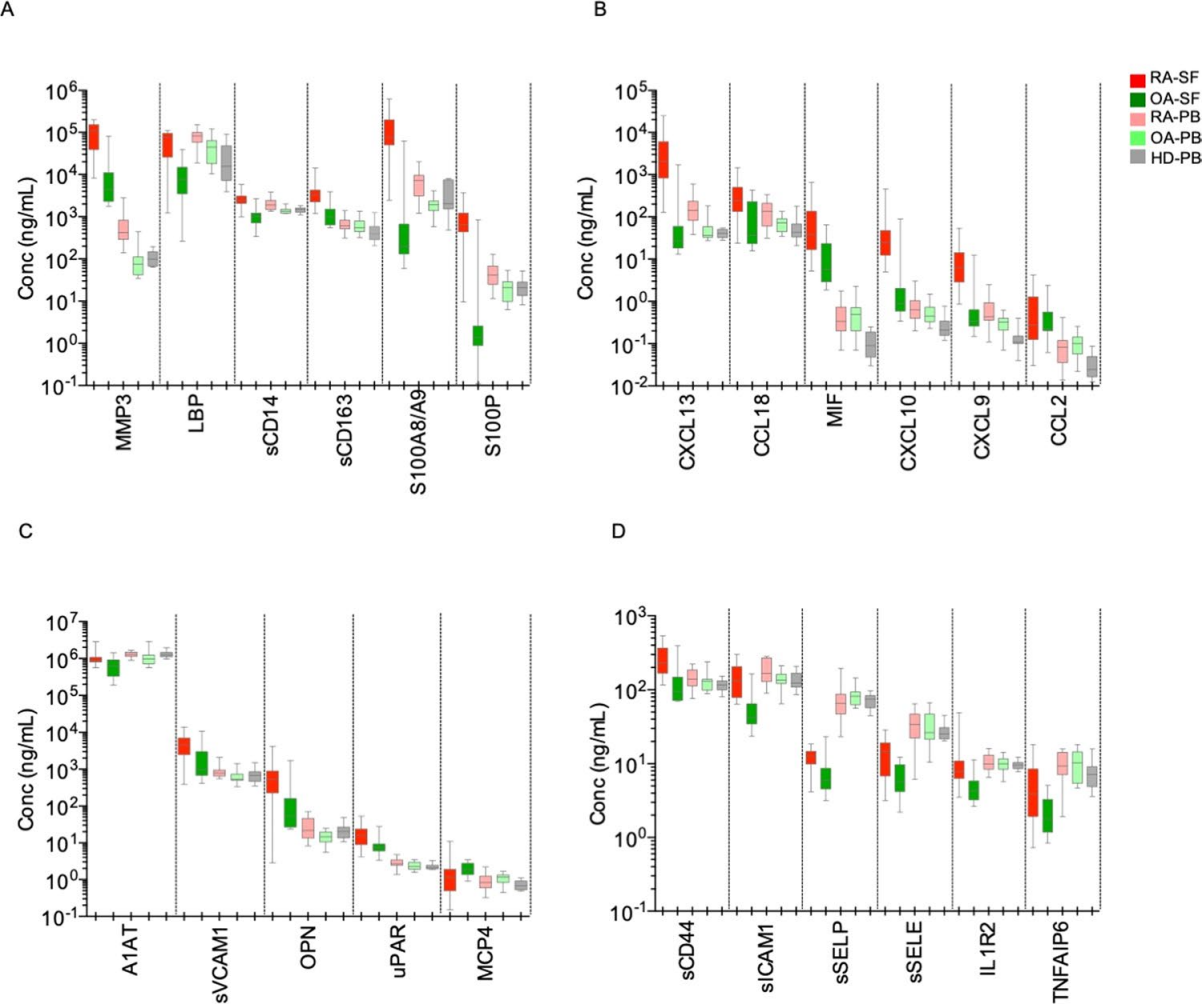
Validation of 23 secreted proteins in RA-SF and corresponding sera indicate their predominant production in the joint. (**A** and **B**) Inflammatory response molecules that differentiated RA-SF from OA-SF (exception: CCL2), and RA-serum from HD-serum. These proteins were elevated in RA-SF when compared to RA-serum (exception: LBP) and obviously diluted and/or neutralised in sera. (**C** and **D**) Inflammatory molecules that differentiated RA-SF from OA-SF and showed moderate or no dilution in sera. Protein concentrations were measured in paired samples of synovial fluid (SF) and serum from RA (n = 18) and OA (n = 15) patients as well as in serum from healthy donors (HD; n = 14). RA-SF: red; OA-SF: green; RA-serum: light red; OA-serum: light green, HD (healthy donors: grey).

In total, 7 molecules revealed higher concentration in RA compared to OA in both, SF and serum, and also in serum from RA when compared to HD (Table 1, Fig. 7). This selection included sCD14, S100A8/A9, S100P, LBP, CXCL13, MMP3 and CCL18. All revealed good correlation between SF and serum concentrations and correlated with DAS28-ESR (supplementary table 9 and supplementary figure 11). With exception of MMP3, their concentrations in OA-SF were lower or equal to those in sera from HD (Fig. 7). This suggests that these mediators produced in the inflamed joints of RA patients not only confirm the concept of molecular pattern selection and interpretation but may also serve as serum markers of RA synovitis activity.

## Discussion

In this study we demonstrated that the dominant changes in long-standing RA-ST consist of infiltrating monocytes/macrophages with activation patterns that correspond best to activation induced by microbial stimuli. Taking advantage of the many cell type and stimulation specific transcriptomes, which we generated on RA-ST, OA-ST and various immune cells in the past and extended with data specifically retrieved from the large GEO data collection, we performed a comprehensive transcriptome analysis of RA and OA synovium and showed that long-standing RA-ST revealed patterns that largely and best overlapped with patterns induced with bacterial and fungal activation of myeloid cells. Patterns of activated B- or T-cells in RA-ST suggested that these lymphocytes respond to but do not initiate monocyte/macrophage activation. Part of these myeloid patterns were proinflammatory chemokines and cytokines and thus, obviously contribute to the attraction of T- and B-cells in RA synovium. In contrast to RA, OA-ST displayed weak gene-patterns of normal tissue macrophages, which seems to reflect response to tissue damage but without development of innate inflammatory patterns. These results were confirmed for proteins secreted and shedded by activated myeloid cells at the site of inflamed synovial tissue in patients with long-standing RA. Chemokines like CXCL9, CXCL10, CXCL13, CCL18 and especially alarmins like S100A8/A9 and S100P, which were among the top ranks of RA-ST, demonstrated that their concentration tremendously declined from a high level in the synovial fluid to a much lower level in serum. The importance of these results was supported by correlation of the concentrations of these proteins between SF and serum, and correlation of both with the disease activity score DAS28.

There is increasing evidence that monocytes/macrophages are key players in initiating and driving chronicity of joint inflammation in RA. Some of the inflammatory molecules produced by monocytes/macrophages have been already included for quantitative assessment of disease activity in RA and are part of the multi-biomarker test Vectra^35^. Predominant infiltration of macrophages into RA synovium is known from histological scoring and was recently affirmed by single cell RNA-sequencing from RA synovial tissues^23,24,36–38^. In our previous study, we could demonstrate that RA monocytes have an increased turnover, characterised by more rapid production and release from bone marrow into the blood, reduced time in circulation and pronounced recruitment into synovium^34^. Furthermore, CD14^++^CD16^+^ monocytes with an intermediate-like phenotype were a dominant subset in RA synovial fluid and seem to shed surface molecules and secret proinflammatory cytokines as a sign of activation after infiltration into the joint^34^.

Here, monocyte/macrophage dominance in RA-ST transcriptomes from patients with long-standing disease was confirmed and the identified inflammatory pattern was deciphered by comparison with many different transcriptomes of monocytes activated by i) exogenous triggers like bacteria, fungi, viruses, and stimuli with pathogen-associated molecular patterns (PAMP) like LPS, zymosan, NOD2-ligand (muramyl dipeptide), TLR2/1-ligand (triacylated lipopeptide), or by ii) endogenous inflammatory mediators including S100A8, TNF, IFNγ, IL15 and IL1β. These inflammatory mediators included many cytokines/chemokines with some of them produced after bacterial and fungal stimulation. For example, production of IL15 and IL1β depended on innate triggers and these cytokines alone induced a stimulation pattern that correlated with bacterial/fungal stimulations but was weaker in intensity. Significant differences, however, were observed between these bacterial/fungal triggers and viral stimulation. Direct stimulation of monocytes *in vitro* with H5N1 revealed an IFNα imprint, which overlapped in part with those induced by bacterial stimulation in monocytes/macrophages. The significant overlap between the IFNα and IFNγ induced patterns in monocytes, as we showed here and in our previous study, may explain the observed IFN imprint in RA-ST that is a result of IFNγ production, predominantly by activated T-cells and not IFNα by plasmacytoid DCs^30^.

Although recent RNA-sequencing analyses of monocyte activation by immune complexes (plate coated IgG – (cIgG) that mimics deposition of IgG-IC) suggested their contribution to inflammation, its pattern was clearly different compared to the RA-ST pattern in our study^39,40^. Using these sequencing data with 77 genes overlapping to our top 100 RA-ST genes and applying the cumulative sum score of the RNA-sequencing comparison between cIgG treated monocytes and unstimulated controls, some genes were also increased but the overall score of cIgG alone did not show a substantial overlap with gene regulation of the top 100 RA-ST genes. Only RNA-sequencing of the LPS-stimulated monocytes or LPS and cIgG co-stimulation revealed a relevant overlapping with RA-ST gene regulation (supplementary figure 12).

Recently Zhang *et al*. characterized by scRNA-sequencing four different monocyte subsets (SC-M1 to SC-M4) in RA-ST and demonstrated that the activated phenotype was more abundant in lymphocyte-rich RA-ST than in OA-ST or lymphocyte-poor RA-ST^41^. This supports the assumption that the strength of monocytes/macrophages activation is important for RA subtypes that differ in disease activity and response to treatment^42–46^. Mapping their transcripts, which characterized the 4 different macrophage types in RA-ST (SC-M1 to SC-M4) with the 203 reference transcriptomes applied in our study, revealed that SC-M2 perfectly overlapped with differentiated Mf in OA-ST and that SC-M1, SC-M3 and SC-M4 phenotypes contained transcripts that in part corresponded with innate stimulation patterns in monocytes/macrophages. In fact, the pattern in SC-M1 was also associated with “LPS stimulated macrophages” and “monocytes treated with LPS” by immunologic gene sets in the MSigDB as a part of GSEA and was named IL-1β^+^ proinflammatory monocytes^41^. This naming according to one candidate (IL-1β^+^) instead of the suggested trigger (LPS) may reflect that scRNA-sequencing of inflammatory synovial monocytes in RA was not sensitive enough to detect the complete proinflammatory pattern and thus gave comparatively weak indications for a more specific functional annotation in RA (supplementary figure 13). This was also stated by these authors, who could find high expression of chemokines in RA-ST only when analysing bulk RNA-sequencing data of the monocyte population, which emphasised that the limited depth of scRNA-sequencing information requires the investigation of whole populations for confirmation of newly identified subsets.

As we showed in this study, the approach of pattern matching with reference transcriptomes and reference comparisons is reasonable and valid. Furthermore, microarray profiles seem to provide at this time much more depth of transcriptional information, higher dimensionality and less susceptibility to technical and biological artefacts. This is an essential advantage for the application as reference transcriptomes and for robust pattern discovery. Our analysis also suggests that many functional gene expression patterns are sufficiently dominant and specific to be recognized in complex data sets with sufficient depth of information, even when analysing whole tissue transcriptomes. It may even provide reference information to characterize scRNA-sequencing data as shown in the supplementary figure 11 and discussed by Zhang *et al*. or others^41,47^. Thus, investigating the extensive data repertoire collected by microarray transcriptome studies for functional patterns of known triggers in diseases of unknown origin like RA can inspire etiopathogenesis research in chronic inflammation.

Interestingly, improvement of arthritis upon fasting is a well-known observation and we could recently associate this improvement with a reduction in monocyte turnover, suggesting that gut microbiota derived triggers may exist in RA^48,49^. The highly elevated levels of these “innate triggering-associated” proinflammatory chemokines in synovial fluid compared to blood raises the question, how this triggering can develop especially in inflamed joints and how fasting as a dietary and gut microbiota influencing intervention can actively decrease or suppress inflammation. While the role of microbial DNA detection in arthritic joints by sequencing produces inconclusive results, spreading of microbial antigens from the gut is alternatively discussed and probably the most likely mechanism^50,51^. Furthermore, searching and finding microbial triggers also in early-RA, or at the disease stage when adaptive immunity does not dominate disease pathogenesis, should facilitate implementation of effective strategies for achieving clinical remission and preventing development and progression of early RA.

Although the search for pathogen related triggers in RA remains and might be related not only to infections but also to bacterial and fungal dysbiosis on mucosal surfaces, in this study we showed differences between the pathogen-like inflammatory response in RA and reaction upon “wear and tear” pathology mechanisms in OA, two joint diseases that require better understanding of pathophysiology to develop causal therapies.

## Methods

### Patients’ characteristics

Synovial tissue samples were obtained from RA (n = 10) and OA (n = 10) patients during joint replacement surgery (synovectomy) by macroscopic selection of highly inflamed and vascularised nonfibrotic villas. Synovitis scores and histological evaluation of cellular composition was performed as previously described and is provided in supplementary table 8^36^. Macroscopically healthy joints of tissue donors (n = 10) ≥8 hours post mortem were selected as described earlier^52^. Joint trauma samples were collected during arthroscopic intervention after injuries. After removal tissue samples were frozen and stored at −70 °C. RA patients were classified according to the American College of Rheumatology criteria valid in the sample assessment period and OA patients were classified according to the respective criteria for OA.

Paired samples of synovial fluid and serum from RA (n = 18), OA (n = 15) and sera from healthy donors (n = 14) were utilised for protein analysis. RA patients were defined by ACR criteria and joint destruction was confirmed radiographically. The group of healthy donors had no signs of inflammation and were not receiving medications. All RA patients were treated with methotrexate, low dose corticosteroids and/or non-steroidal anti-inflammatory drugs (NSAIDs) but no biologics. Patients’ characteristics are summarized in supplementary table 8. Patients were not involved in the design, or conduct, or reporting, or disseminating our research. All participants submitted a written consent before samples were collected. The study was approved by the Ethics committee of the Charité Universitätsmedizin Berlin, and all experiments were performed in accordance with relevant guidelines and regulations.

Calculation of Disease activity score 28 (DAS28) included erythrocyte sedimentation rate as blood marker of inflammation (ESR), together with the number of tender and swollen joints and patient global health assessment. DAS28-ESR ≤ 3.2 indicates low, >3.2 and ≤5.2 moderate and >5.2 high disease activity.

**RNA isolation, Affymetrix GeneChip hybridization, statistical and functional analysis of microarray data** are included in the supplementary file.

### Statistical and functional analyses of microarray data

Analysis with the BioRetis database (www.bioretis.com), consisted of MAS5.0 pair-wise comparison statistics, was performed to select differentially expressed probe-sets as previously described^30,53–55^. Probe-sets (Affymetrix-IDs) of genes were selected as differentially expressed if they 1) revealed either increased or decreased expression in at least 50% of all pair-wise comparisons between RA (n = 10) and OA (n = 10) samples, and 2) if they showed more homogeneous expression in the experimental when compared to baseline group and increased or decreased expression in 30% of pair-wise comparisons as previously described. Selection of probe-sets included signal log ratio (SLR) t-tests with Bonferroni correction for multiple testing^53^.

### Functional analyses of microarray data

**MeV** (MultiExperiment Viewer, version 4.0, MA, USA) was applied for hierarchical clustering of signal and correlation coefficient matrices.

**Qlucore** (Lund, Sweden) was applied for principal component analyses (PCA) of samples and variables.

**Gene set enrichment analyses (GSEA)** (The Broad Institute/MIF, USA) was performed with pre-processed datasets, exactly with 2019 differentially expressed probe-sets identified in pair-wise comparisons between RA and OA synovial tissue transcriptomes. Although this step affects the enrichment score statistics included in GSEA (ES-enrichment score, NES-normalized enrichment score, NOM-nominal p-value, FDR-false discovery rate q-value, and FWER-familywise-error rate p-value) we performed this step since our criteria for selection of genes for analyses are far more stringent than those from GSEA^56^. The GSEA was run with permutation of phenotype for 1000 times, weighted enrichment statistics, log2 ratio of classes as a metrics for ranking genes, and with a minimal gene set size of 10. In total, the top 20 pathways enriched in RA and OA were selected and presented in supplementary table 2.

**Ingenuity pathway analysis (IPA)** was applied to assign the RA and OA profiles from synovial tissues to distinct molecular networks and canonical signalling pathways (IPA, Qiagen Redwood City, USA).

**DAVID functional annotation tool** was applied to annotate differentially expressed probe-sets to biological processes (BP) and cellular components (CC) determined by gene ontology (GO)^57^.

### Functional analysis of RA and OA transcriptomes from synovial tissue (ST) performed with reference transcriptomes

Our own (n = 42) and public data (n = 140) from Gene expression omnibus (GEO) were used for selection of reference transcriptomes. Differentially expressed genes in comparisons between RA- and OA-ST microarrays were utilized for mapping with these reference transcriptomes. In total, 38 Affymetrix microarrays of 12 data sets (each represented with 3 or 4 different microarrays) were applied for analysis of cell infiltration in RA- and OA-ST by reference transcriptomes (supplementary table 4). These arrays included (1) synovial fibroblasts (SFbl; n = 4), (2) endothelial cells (Endo; n = 4), (3) platelets (Plt; n = 3), (4) CD19^+^B-cells (n = 3), (5) CD4^+^T-cells (n = 3), (6) CD8^+^T-cells (n = 3), (7) CD56^+^NK-cells (n = 3), (8) CD1^+^DC (n = 3), (9) CD14^+^ monocytes (Mo; n = 3), (10) macrophages (Mo differentiated for 3 days *in vitro* into macrophage (Mf); n = 3), (11) Mf from RA synovial fluid (RA-SF-Mf; n = 3) and (12) CD15^+^ granulocytes (n = 3)^25–31^.

For analysis of immune cell activation, 182 reference transcriptomes from 64 data sets were applied. Beside the above mentioned 38 arrays, the additional 144 reference transcriptomes were included. Out of these 64 data sets, 14 were generated in our Affymetrix core facility and 50 were selected from GEO repository. Each data set contain 2-4 microarrays. The reference transcriptomes belong to the HG-U133A or HG-U133 Plus 2.0 series and are summarized in supplementary table 5. They portrayed activation and differentiation of B-cell, T-cells and monocytes and included following data sets:

(i) B cells: naïve-B cells, memory-B cells, germinal centres B-cells and plasma cells^58^.
(ii) T-cells and their activation profiles: Th1, Th2, Th17, naïve T-cells, regulatory T-cell, γδT-cells and activated Th1, Th2 and γδT-cells^33,59,60^.
(iii) Myeloid cells (Mo, Mf, DC) and their activation triggered by microbial and inflammatory stimuli such as:

(A) **Bacterial:** (1) *Mycobacterium tuberculosis*, (2) *Staphylococcus aureus*, (3) *Lactobacillus rhamnosus* LGG, (4) NOD2L stimulation (muramyl dipeptide), (5) TLR2/1 L stimulation (triacylated lipopeptide), (6) *Francisella novicida*, and (7) *Chlamydia pneumoniae*^61–66^.
(B) **Fungal:** (1) Zymosan A and (2) *Aspergillus fumigatus*^67^.
(C) **Viral:** (1) H5N1 influenza and (2) live attenuated viral strain of yellow fever vaccine^55,68^.
(D) **Cytokines:** TNF, LPS IFNα, IFNγ, IL1β, IL4, IL10, IL15^30,54,69,70^.
(E) **Alarmin:** S100A8^69^.
(F) ***In vitro*** **polarisation** of monocytes to M1-Mf or M2-Mf were performed with LPS + IFNγ and IL4, respectively^71^.

To harmonize the data from different studies, all reference transcriptomes were integrated into the BioRetis database, were quantile normalised and were subsequently applied for co-expression analysis. Pearson correlation coefficients were calculated between the probe-sets differentially expressed in RA-ST and OA-ST on the basis of the signals in the 182 different reference transcriptomes. Hierarchical clustering of this gene-to-gene correlation matrix was performed by applying Euclidean distance and average linkage as an agglomeration rule, as previously described^34^.

In 4 data sets, different stimuli were used more than once in a single experiment and each of them was compared with control samples. To notice differences between each stimulus and control array, the control samples were placed first, followed by the corresponding stimulus in the heatmap (Fig. 4 and supplementary figure 6), which resulted in the presence of control arrays more than once for these experiments. Thus, altogether 182 different reference transcriptomes were applied for the analysis, and some control samples were used more than once, which resulted in 203 arrays in the heatmap. In the following experiments, arrays of control samples were used more than once: 1) TNF, LPS, IFNα2a and IFNγ stimulation, 2) IL4, IL10 and IL15 stimulation, 3) NOD2L and TLR2/1 L activation and 4) IL1β and S100A8 stimulation. The organisation of the 203 arrays is provided in supplementary table 5.

**An overview of ELISAs and multiplex bead assays** for protein detection and statistical test for data analysis is provided in the supplementary file.

### Statistical analyses of protein data

GraphPad Prism V-6.0b was used for statistical analysis of data obtained by measuring soluble markers in SF and serum. Concentrations of measured markers were compared either by Mann-Whitney U-test (RA-SF and OA-SF) or by Kruskal-Wallis test coupled with Dunn’s multiple comparisons test (RA, OA and HD serum). P-values < 0.05 were considered significant. Spearman correlation coefficients were calculated for measuring statistical dependence between 1) concentrations of soluble markers in SF and serum, 2) concentrations of soluble markers in SF and clinical data and 3) concentrations of soluble markers in serum and clinical data.

### Data availability

Own Affymetrix gene array data (n = 42) are accessible in GEO with following numbers: GSE55235, GSE58173, GSE51997, GSE38351.

## Supplementary information

Supplementary information.

Supplementary Table 1

Supplementary Table 2

Supplementary Table 3

Supplementary Table 4

Supplementary Table 5

Supplementary Table 6

Supplementary Table 7

Supplementary Table 8

Supplementary Table 9
