# Supplementary material for: Synovial tissue transcriptomes of long-standing rheumatoid arthritis are dominated by activated macrophages that reflect microbial stimulation
Source: Sci Rep. 2020 May 13;10:7907. doi: 10.1038/s41598-020-64431-4 (PMC7220941; doi:10.1038/s41598-020-64431-4)
Supplement: Supplementary file 1 — Supplementary information. [file 41598_2020_64431_MOESM1_ESM.pdf]

Synovial tissue transcriptomes of long-standing rheumatoid arthritis are dominated by activated macrophage that reflect microbial stimulation

Biljana Smiljanovic[1], Andreas Grützkau[2], Till Sörensen[1], Joachim R. Grün[2], Thomas Vogl[3], Marc Bonin[1], Pascal Schendel[1], Bruno Stuhlmüller[1], Anne Claussnitzer[1], Sandra Hermann[1], Sarah Ohrndorf[1], Karlfried Aupperle[1], Marina Backhaus[1], Andreas Radbruch[2], Gerd R. Burmester[1], Thomas Häupl[1*]

[1]Department of Rheumatology and Clinical Immunology, Charité Universitätsmedizin, Berlin, Germany

[2]Deutsches Rheuma-Forschungszentrum Berlin (DRFZ), a Leibniz Institute, Berlin, Germany

[3]Institute of Immunology, University of Münster, Germany

Supplementary file:

Methods:

RNA isolation, Affymetrix GeneChip hybridization and quality control for gene-expression analyses

Tissue homogenization, total RNA isolation and treatment with RNAase-free DNase I were performed according to the manufacturer's instructions (Qiagen, Hilden, Germany). RNA preparation, quality controls and array hybridization were performed as previously described[1,2]. In brief, total RNA was extracted (RNeasy Mini Kit, Qiagen, Hilden, Germany), and its quality was assessed by Agilent 2100 Bioanalyzer (Agilent Technologies, Waldbronn, Germany) and quantified by NanoDrop ND-1000 spectrophotometer (NanoDrop Technologies, Wilmington, DE, USA). After reverse transcription of 3-5 µg of total RNA (Ambion, Austin, TX, USA) and generation of cRNA probes (Enzo RNA Transcript Labeling kit; Affymetrix), 15 µg of biotinylated and fragmented cRNA was hybridized to Affymetrix HG-U133A arrays, which cover >14.500 well-characterized human genes and around 22.000

probe sets. After washing and fluorescence labelling, arrays were scanned (Affymetrix GeneChip Scanner 3000).

Calculation of scores that quantify differential expression.

For comparative analysis, pairwise comparisons between two arrays with the MAS5.0 algorithms generated for each probe set the signal log ratio (*SLR*) and the p-value to provide the call for significant increase or decrease of expression (true=1, false=0). Both, SLR and call were used for calculation of the probe set (*ps*) specific score for increased expression:

$$score_{ps_{inc}} = \frac{\sum_{i=1}^{n} SLR_{ps}}{n} \times \frac{\sum_{i=1}^{n} significant\ increase_{ps}}{n}$$

or decreased expression:

$$score_{ps_{dec}} = \frac{\sum_{i=1}^{n} SLR_{ps}}{n} \times \frac{\sum_{i=1}^{n} significant\ decrease_{ps}}{n}$$

with n representing the number of pairwise comparisons for one group comparison (e.g. comparison between 10 RA-ST with 10 OA-ST arrays generates 10x10=100 pairwise comparisons for each probe set). The scores were applied to rank the genes and to perform cumulative scoring by stepwise summing up the scores of each gene in the list starting with the top rank.

Quantitative assessment of cell type-related activation and differentiation processes.

To estimate the regulation of genes as a consequence of cell type related activation and differentiation processes, reference transcriptomes of defined stimulation experiments were compared to their cell type specific controls. In detail, the scores for each gene were calculated as described above for the comparison between RA-ST and OA-ST transcriptomes and between reference transcriptomes and their controls (n=35, see also supplementary table 6 for details of the arrays used in the comparisons). The top 100 genes were selected out of 1010 and 345 up-regulated probe sets that determined the whole RA-ST profiles and the RA-ST profile of secreted molecules, respectively. The top 100 genes with the highest score values in RA-ST were selected and ranked from 1-100 (abscissa in figures 5, 6 and supplementary figure 7). For these genes, the scores determined in the 35 reference comparisons were cumulatively added (ordinate in these figures) and displayed for each reference comparison as indicated in the

legend. Correspondingly, the top 100 genes with the highest score values in OA-ST were ranked from 1-100 and score values of the 35 comparisons were cumulatively added (supplementary figure 4 presents top genes from the overall OA-ST profile and supplementary figure 8 presents genes of secreted proteins in OA-ST).

When performing quantitative assessment, two additional reference comparisons were included: 1) stimulation of neutrophils and 2) discrimination of native from *in vitro* proliferating fibroblast. Neutrophils revealed a partial overlap with monocyte patterns when using transcript expression signals of the reference transcriptomes. To clarify, whether both types of phagocytes are contributing to the RA-ST specific pattern, we tested also gene regulation by exogenous triggers and included the scores induced by *S.aureus* stimulation of neutrophils. To gain additional information about synovial fibroblasts for explaining their contribution in OA, the comparison between RA and OA synovial fibroblasts was insufficient since these samples were analysed after their *in vitro* proliferation, which appeared as dominant process that largely masked initial differences between samples. Based on the well-established proliferative stroma cell (fibroblast) response in RA by histology, we tested whether the OA-ST specific pattern may be more represented by normal synovial tissue than by proliferating fibroblasts[3]. For this reason, we performed an additional comparison between synovial tissues of healthy donors early post mortem and proliferating synovial fibroblast in culture (reference comparison 2 in supplementary table 6).

Scoring of RA-ST related transcriptional changes in reference comparisons

The relevance of RA-ST related changes in the reference comparisons was not only estimated by determining the intersecting set of genes between the top 100 RA-ST genes and each of 35 comparison but also by determining the top 100 genes in each of the 35 reference comparisons. For each top 100 gene set in the 35 reference comparisons, RA-ST scores were selected and shown as the cumulative sum of RA-ST scores in supplementary figure 5. In these graphs, the ranks on the abscissa represent genes, that are specific for each of the different reference comparisons. The list of these top 100 genes in each reference comparison is provided in supplementary table 7, Worksheet-E.

For protein analysis by ELISA and multiplex bead assays the matched samples of synovial fluid and serum from RA (n=18) and OA (n=15) patients and sera from healthy donors (n=14)

were examined for 27 markers according to manufacturer's instructions. The following ELISAs were used: sCD14, sCD163, CXCL13/BLC, IL1R2, Osteopontin (OPN (SPP1)), and uPAR (all from Quantikine R&D Systems, Inc., Minneapolis, USA); Human CCL18/PARC and CD44 (both from Cell Sciences, MA, USA); A1AT and CCL13 (MCP4) (both from Abcam, Cambridge, UK); MMP3 (GE Healthcare, UK), S100P (CircuLex, CycLex Co., Ltd. Japan), TNFAIP6 (Cusabio, China), LBP (Elabscience, Wuhan, China), S100A8/A9 (in-house ELISA kindly provided by Prof. Dr. T. Vogl, Institute of Immunology, University of Münster), IFNα (PBL Interferon source, USA). The soluble form of adhesion molecules sICAM1, sVCAM1, sSELE and sSELP were measured by Human adhesion molecule multiplex kit (R&D Systems, Inc., Minneapolis, USA). Cytokines including TNF, IFNγ, IL8, CXCL9 (MIG), CXCL10 (IP10), MIF and CCL2 (MCP1) were measured by Bio-Plex Pro Assays (Bio-Rad Laboratories, Inc., USA).

References:

1 Biesen, R. *et al.* Sialic acid-binding Ig-like lectin 1 expression in inflammatory and resident monocytes is a potential biomarker for monitoring disease activity and success of therapy in systemic lupus erythematosus. *Arthritis and rheumatism* **58**, 1136-1145, doi:10.1002/art.23404 (2008).

2 Smiljanovic, B. *et al.* Defining TNF-alpha- and LPS-induced gene signatures in monocytes to unravel the complexity of peripheral blood transcriptomes in health and disease. *J Mol Med (Berl)* **88**, 1065-1079, doi:10.1007/s00109-010-0648-8 (2010).

3 Krenn, V. *et al.* Synovitis score: discrimination between chronic low-grade and high-grade synovitis. *Histopathology* **49**, 358-364, doi:10.1111/j.1365-2559.2006.02508.x (2006).

4 Zhang, F. *et al.* Defining inflammatory cell states in rheumatoid arthritis joint synovial tissues by integrating single-cell transcriptomics and mass cytometry. *Nature immunology* **20**, 928-942, doi:10.1038/s41590-019-0378-1 (2019).

Supplementary figures and tables:

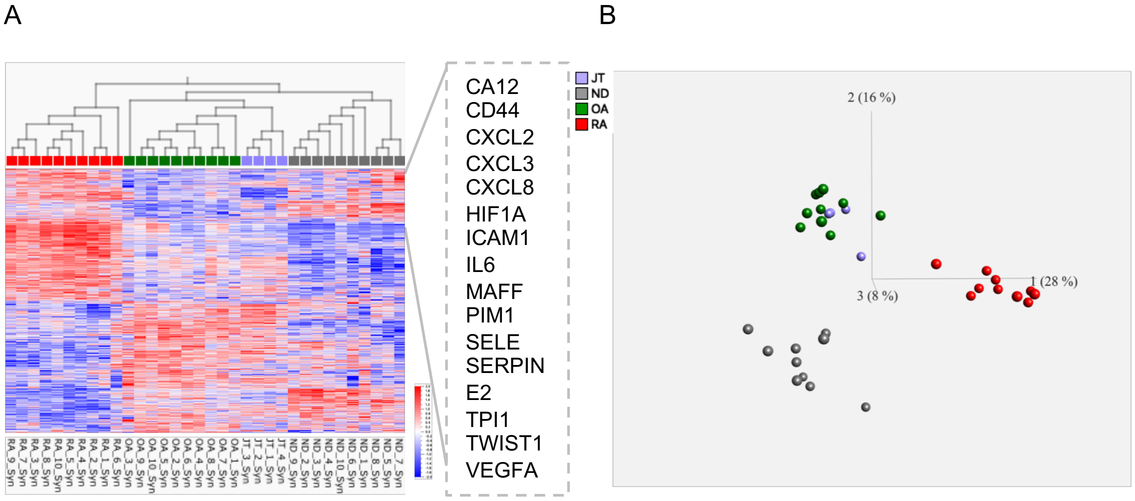

Supplementary figure 1. RA synovial tissue transcriptomes differentiated not only RA from OA synovial tissue, but also RA from joint trauma (JT) and post mortem tissue donor (ND) samples. **(A)** Hierarchical clustering of 2019 Affymetrix probe-sets identified in comparisons between RA-ST (n=10) and OA-ST (n=10) together with synovial tissues obtained post mortem (ND, n=10, samples from our own collection) and from patients after joint trauma (n=4; GSE12021). **(B)** PCA of the same synovial tissue samples clearly separated RA-ST transcriptome profile from OA-ST and JT-ST. Although hypoxia induced alterations in synovial tissue transcriptomes of post mortem samples demonstrated some inflammatory profile, which partially overlapped with RA-ST, the ND-ST samples clustered with OA-ST and JT-ST and showed a distance from RA-ST by PCA. RA-red, OA-green, post mortem ND (gray) and JT (lilac).

A **B** **C**

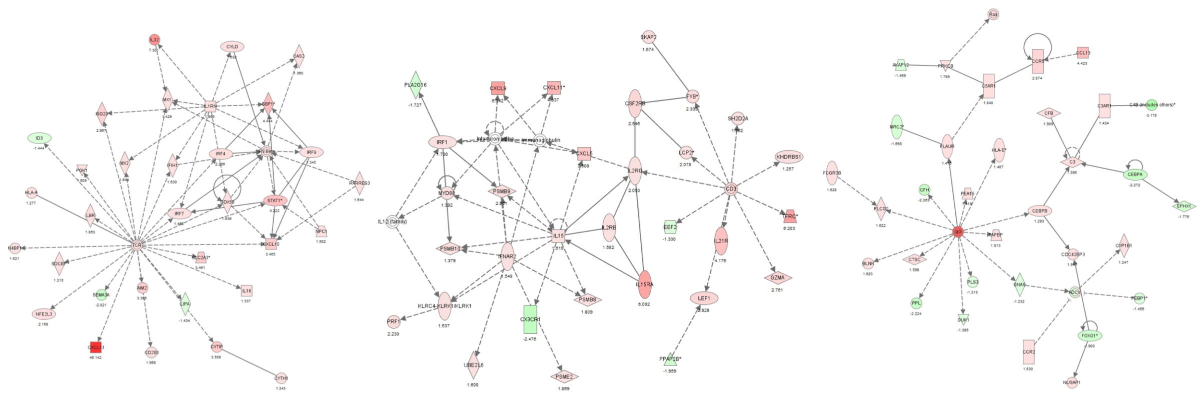

Supplementary figure 2. Molecular networks identified in RA-ST by Ingenuity pathway analysis. The key molecular networks depicted possible interactions between genes differentially expressed in RA-ST when compared to OA-ST **(A-C)**. Up- and down-regulated genes in RA-ST were coloured in red and green, respectively. Numbers below genes indicate fold change difference in comparisons between RA- and OA-ST.

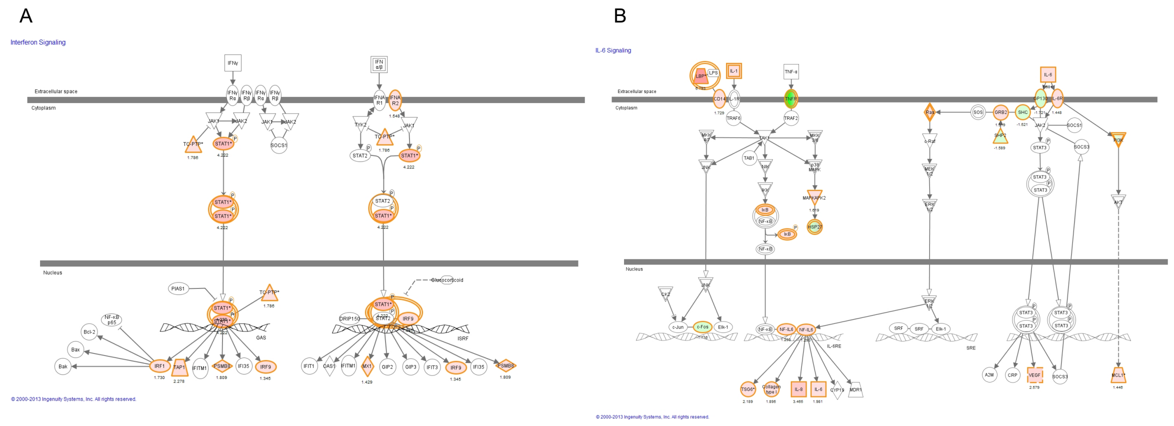

Supplementary figure 3. Signaling pathways identified by Ingenuity pointed to involvement of inflammatory mediators: IFN, TNF, IL1, IL6 and LBP (**A** and **B**), which influenced the inflammatory milieu in synovial tissue from RA patients. Up- and down-regulated genes in RA-ST were coloured in red and green, respectively. Numbers below genes indicate fold change difference in comparisons between RA- and OA-ST.

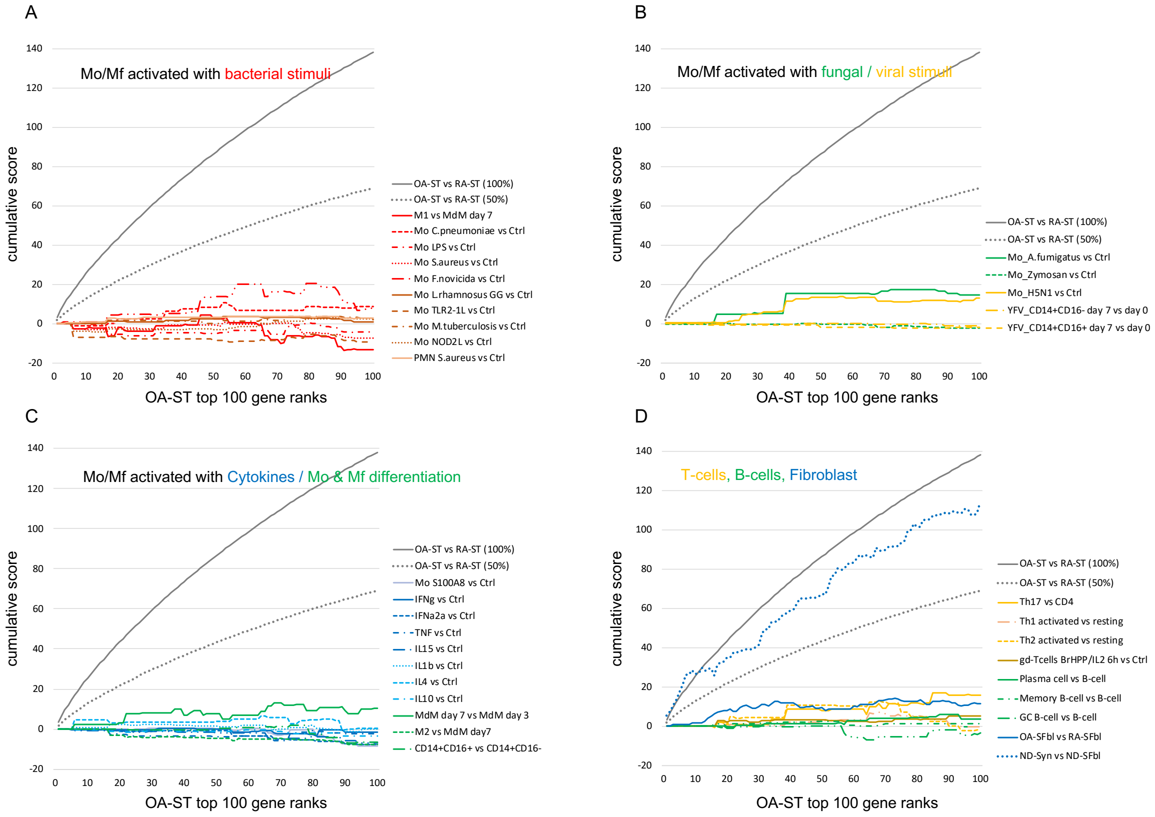

Supplementary figure 4. Quantitative assessments of cell type and stimulus specific activation of the top 100 genes in OA-ST. Out of the 1009 probe-sets up-regulated in OA-ST vs RA-ST, the top 100 genes with the highest score values were selected and ranked by scores. These ranks are represented on the abscissa. The ordinate shows the cumulative sum of scores as it changes with each newly added gene. As target value, the grey line indicates the cumulative sum of scores of OA-ST (100% score) and as auxiliary value, the dotted grey line indicates its 50% value. For the genes on the abscissa, we applied the scores obtained from different comparisons between reference transcriptomes as indicated in the legend to the right and in supplementary table 6. **(A)** presents the cumulative sum of scores for bacterial activation of monocytes/macrophages with LPS and IFNγ (M1-Mf), *C. pneumoniae*, LPS, *S. aureus*, *L. rhamnosus*, *F. novicida*, TLR1/2L, *M. tuberculosis*, NOD2L and granulocytes with *S. aureus*. **(B)** indicates the scoring achieved by fungal and viral activation in monocytes/macrophages induced by *A. fumigatus*, zymosan A, H5N1 influenza virus and yellow fever vaccination in classical ($CD14^{++}CD16^{-}$) and non-classical ($CD14^{+}CD16^{+}$) monocytes. **(C)** shows the scoring from reference comparisons that present cytokine induced activation and differentiation in monocytes/macrophages. It included profiles of TNF, IFNγ, IFNα2a, IL4, IL10, IL15, IL1β and S100A8 stimulation, of macrophage differentiation with CSF2 for 7 days and of non-classical ($CD14^{++}CD16^{-}$) compared to classical ($CD14^{++}CD16^{-}$) blood monocytes. (**D**) outlines

the scoring for activated T-cells, B-cells and synovial fibroblasts (SFbl). Activation of T-cells was determined in comparisons between Th1 activated vs Th1 resting, Th2 activated vs Th2 resting, and Th17 activated vs CD4 T-cells. B-cell profiles were determined in comparisons between plasma-, memory B- and GC-B-cells to naïve B-cells. Profiles of synovial fibroblasts were determined in comparisons of *in vitro* cultured RA-SFbl and OA-SFbl and of native synovial tissue compared with *in vitro* cultured SFbl both from normal joints of tissue donors (collected early post mortem).

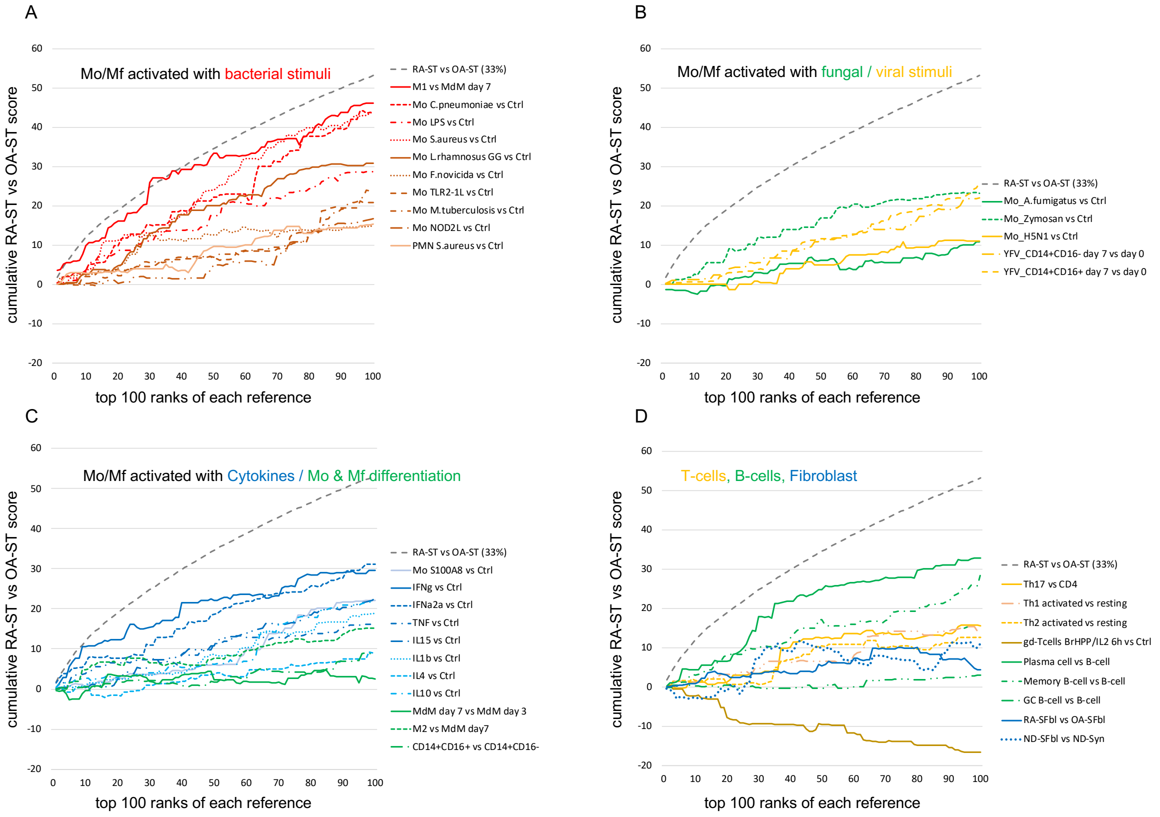

Supplementary figure 5. Performance of RA-ST vs OA-ST scores in the top 100 genes of the reference comparisons. Besides the overlap of RA-ST gene regulation with the top 100 genes especially of bacterial and fungal triggers, we tested the overall performance of RA-ST gene regulation in each of the 35 reference comparisons by selecting for each reference comparison the 100 top candidates (ranks on the abscissa) and calculating the cumulative sum score with the RA-ST scores (ordinate). Similar to figure 5, candidates and corresponding scores are displayed for bacterial (A), viral and fungal (B) and cytokine triggering of monocytes (C) and for activation of other immune cells and fibroblasts (D). The list of reference comparisons is presented in supplementary table 6 and the top 100 genes for each reference comparisons are presented in supplementary table 7, worksheets A-E.

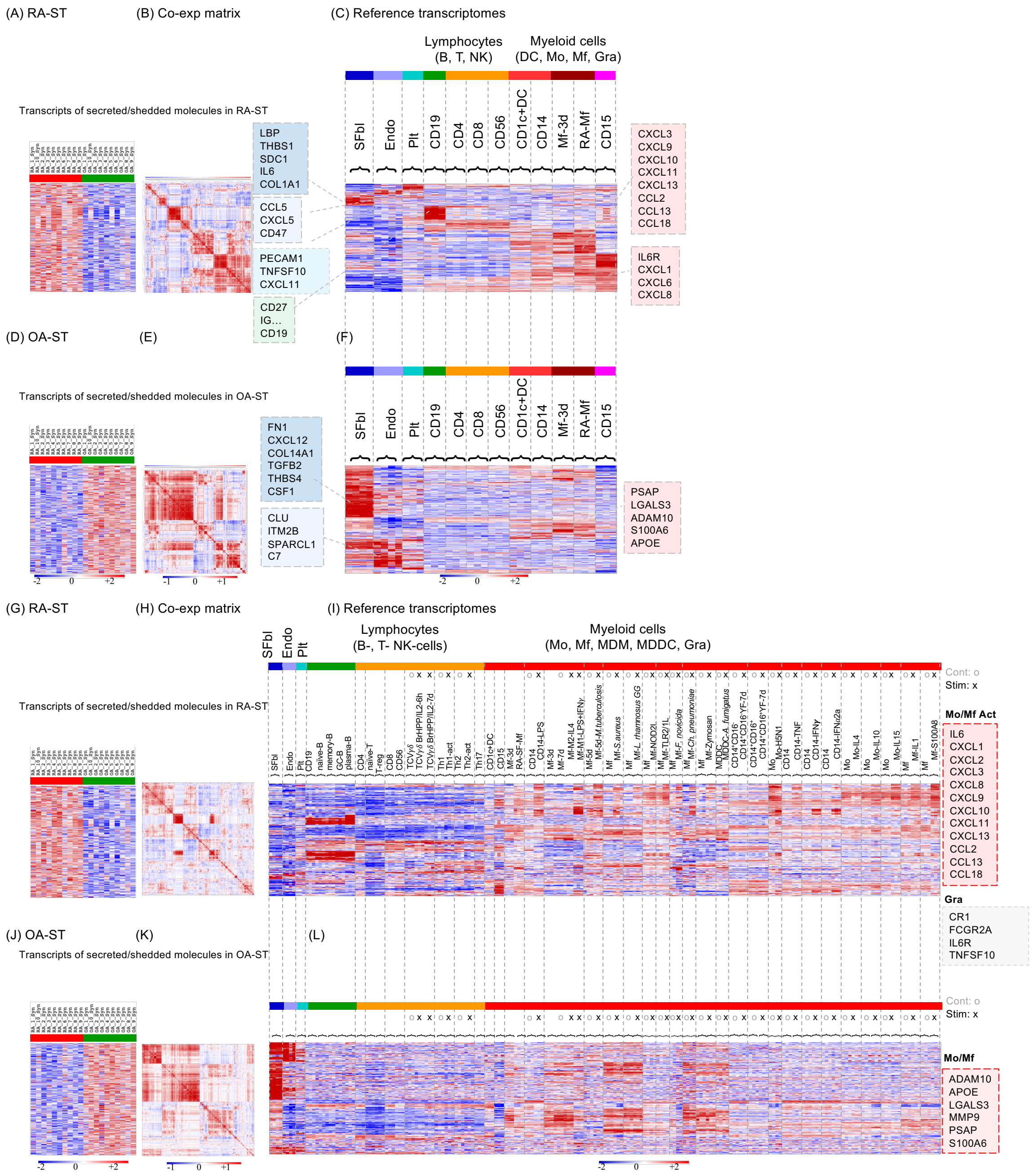

Supplementary figure 6. Gene-patterns of molecules that can be secreted or shedded are predominantly derived from monocytes/macrophages in RA-ST and from synovial fibroblast in OA-ST. Transcripts of secreted and shedded molecules were analysed for infiltration of cells with 38 reference transcriptomes (**A-F**) and for infiltration and activation of cells with 182 reference transcriptomes (**G-L**).

In total, 345 out of 1010 up-regulated probe-sets in RA-ST (**A** and **G**), and 405 out of 1009 probe-sets up-regulated in OA-ST (**D** and **J**), were selected based on gene ontology (GO) annotations for "extracellular space" and "extracellular exosomes" (GO:0005615 and GO:0070062).

Analysis of cell infiltration: similar to figure 3, co-expression matrices (**B** and **E**) were calculated and patterns aligned to expression in RA-ST and OA-ST (**A** and **D**), as well as to expression in the 38 cell type reference transcriptomes (**C** and **F**).

Analysis of cell infiltration and activation: similar to figure 4, co-expression matrices (**H** and **K**) were calculated and patterns aligned to expression in RA-ST and OA-ST (**G** and **J**), as well as to expression in the 182 cell type reference transcriptomes (**I** and **J**).

For colour codes see figure 4.

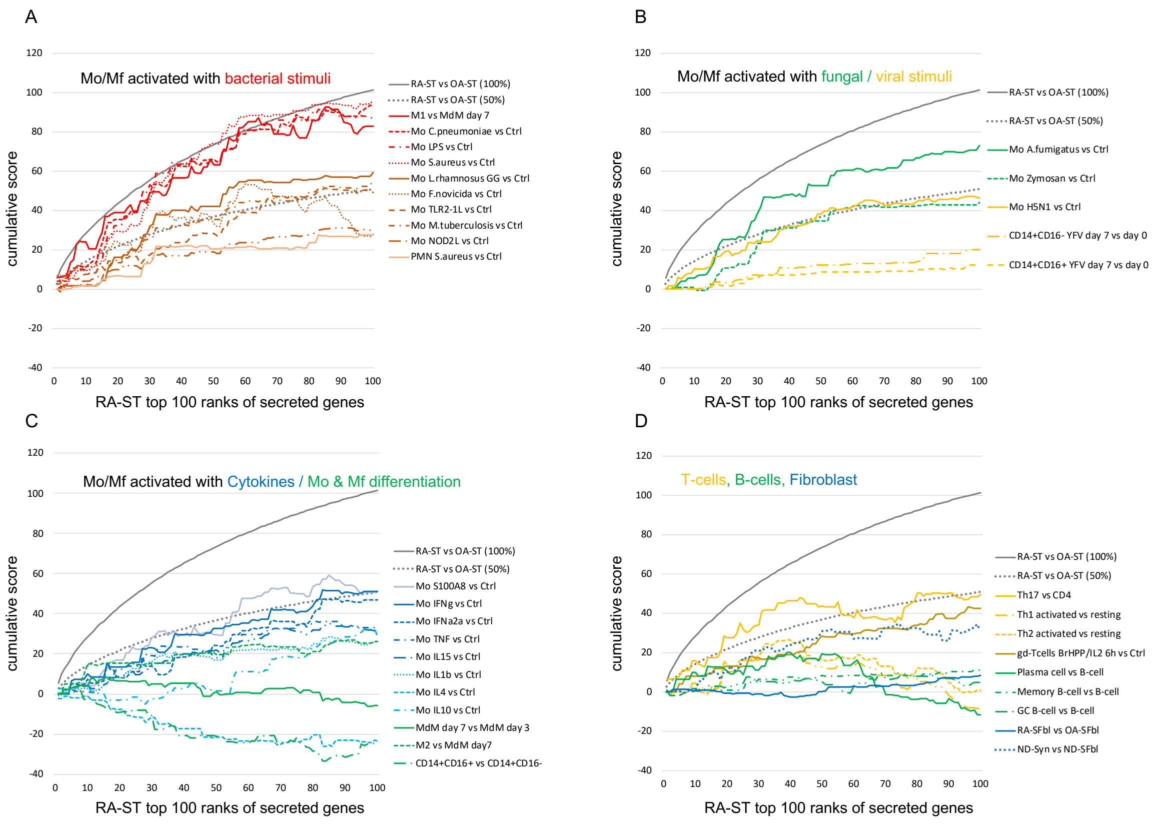

Supplementary figure 7. Quantitative assessment of bacterial, fungal-, viral-, cytokine-induced and cell type specific activation in the top 100 genes encoding secreted/shedded molecules in RA-ST. As described in supplementary figure 6, 345 out of the 1010 probe-sets up-regulated in RA-ST encoded secreted/shedded molecules. Out of these 345 probe-sets, the top 100 genes with the highest score values were selected and ranked by scores. These ranks with gene names are represented on the abscissa. The ordinate shows the cumulative sum of scores as it changes with each additional rank and its gene. As target value, the grey line indicates the cumulative sum of scores for secreted/shedded molecules of RA-ST (100% score) and as auxiliary value, the dotted grey line indicates its 50% value. For the genes on the abscissa, we applied the scores obtained from different comparisons between reference transcriptomes as indicated in the legend to the right and showed in supplementary table 6. **(A)** presents the cumulative sum of scores for bacterial activation of monocytes/macrophages (details provided in figure 6). The cumulative sum of scores for (**B**) fungal and viral activation in monocytes/macrophages induced by *A. fumigatus*, zymosan A, H5N1 influenza virus and yellow fever vaccination in classical ($CD14^{++}CD16^{-}$) and non-classical ($CD14^{+}CD16^{+}$) monocytes. **(C)** The cumulative sum of scores for cytokine induced activation and differentiation in monocytes/macrophages including TNF, IFNγ, IFNα2a, IL4, IL10, IL15, IL1β and S100A8 stimulation, macrophage differentiation with CSF2 for 7 days and

differentiation of non-classical ($CD14^{++}CD16^{-}$) from classical ($CD14^{++}CD16^{-}$) blood monocytes; (**D**) outlines the scoring for activated T-cells, B-cell and synovial fibroblast (SFbl). Activation of T-cells was determined in comparisons between Th1 activated vs Th1 resting, Th2 activated vs Th2 resting, and Th17 activated vs CD4 T-cells. B-cell profiles were determined in comparisons between plasma-, memory B- and GC-B-cells to naïve B-cells. Profiles of synovial fibroblasts (SFbl) were determined in comparisons of *in vitro* cultured RA-SFbl and OA-SFbl and of native synovial tissue compared with *in vitro* cultured SFbl both from normal joints of tissue donors (collected early post mortem).

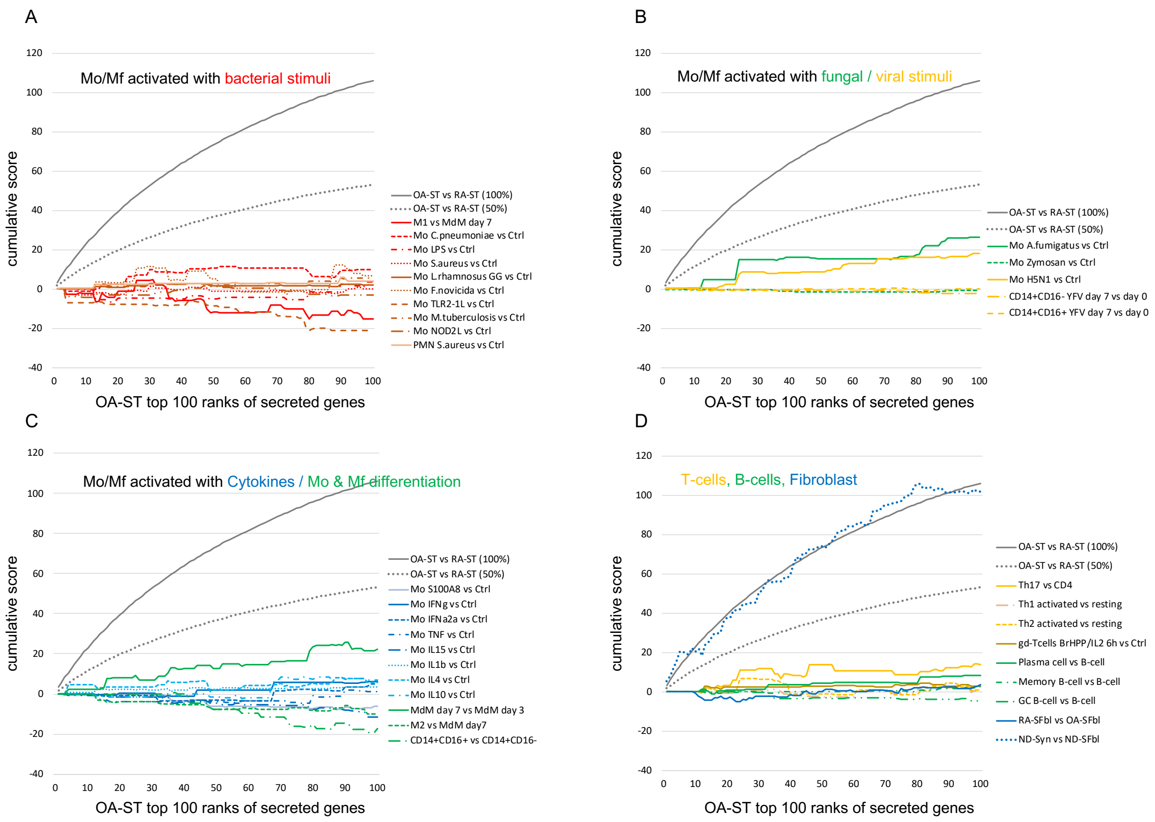

Supplementary figure 8. Quantitative assessment of bacterial-, fungal-, viral-, cytokine-induced and cell type specific activation in the top 100 genes encoding secreted/shedded molecules in OA-ST. This figure corresponds to supplementary figures 4 and 7, with the exception that the 1009 probe-sets up-regulated in OA-ST were reduced to the 405 probe-sets, which encoded secreted/shedded molecules and from these, the top 100 genes with the highest scores were analysed. These top 100 genes in OA-ST were listed in supplementary table 7, worksheet-D.

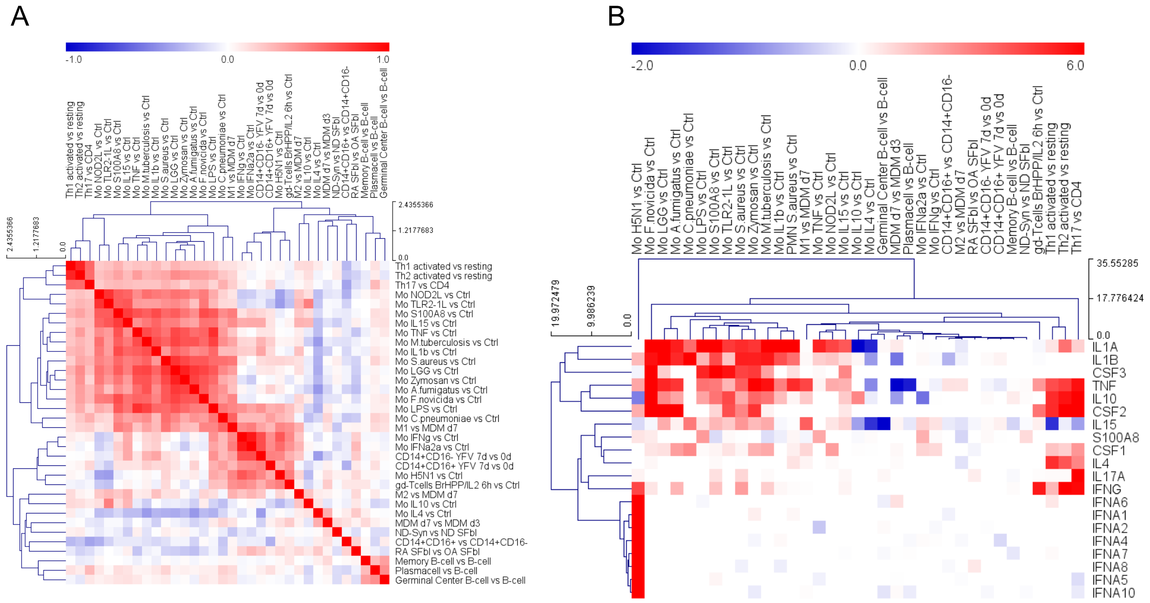

Supplementary figure 9. Correlation of the 35 reference comparisons based on their scores for the top 100 RA-ST genes. (**A**) Pearson correlation between the reference comparisons when using the top 100 RA-ST genes identified the largest cluster with bacterial, fungal, TLR, TNF, IL15 and IL1β stimulations of monocytes, another one with viral and interferon responses and two additional for T cells and B-cells. (**B**) Screening of the cytokines and factors, which were used in the reference comparisons for cell stimulation presented IL15, IL1β and TNF especially after microbial stimulation, IFNα after viral triggering and IFNγ predominantly inducible in stimulated T-cell subsets. These response patterns are in line with the observation of a common correlation cluster for responses to TNF, IL1β, IL15 and microbial components in monocytes in (A). Labels of the reference comparisons are explained in supplementary table 5 and arrays used in each comparison in supplementary table 6.

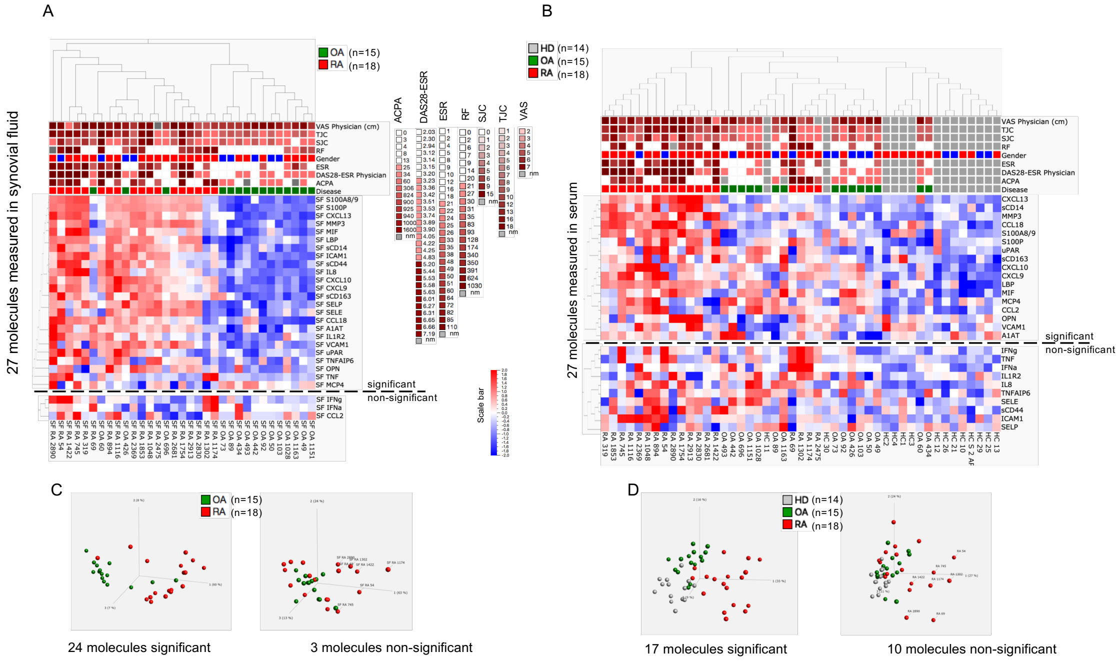

Supplementary figure 10. Confirmation of transcriptome data by quantification of secreted and shedded proteins in paired samples of synovial fluid (SF) and serum from RA (n=18) and OA (n=15) patients and in serum from healthy donors (HD; n=14). Out of 27 measured proteins, 24 reached statistical significance to differentiate RA from OA in SF **(A)** and 17 to differentiate RA, OA and HD in serum (**B**). Statistical significance was determined using Mann-Whitney U-test for calculating p-values in comparison between RA and OA in SF and Kruskal-Wallis test (with Dunn's multiple comparisons test) for calculating p-values in comparison between RA, OA and HD in sera. P-values are indicated in table 1.

Red: RA; green: OA; grey: HD. Protein concentrations were log-transformed, z-normalised and relative differences visualized according to the scale bar colour.

Abbreviations and colours within dendrograms in (A) and (B): ACPA (anti-citrullinated protein antibody): ≤20 coloured in white, >20 coloured gradually from light red (=25) to brown (=1600); DAS28-ESR (disease activity score 28-erythrocyte sedimentation rate): ≤3.2 coloured white, >3.2 to ≤5.1 coloured red, and ≥5.1 coloured brown); ESR (erythrocyte sedimentation rate): ≤20 coloured white, >20 coloured gradually from light red (=21) to brown (=110); gender: female-red and male-blue; RF (rheumatoid factor): ≤20 coloured white, >20 coloured gradually light red (=21) to brown (=1030); TJC (tender joint count): coloured gradually from light red (=1) to brown (=18; maximum value in this group of patients); SJC (swollen joint count): coloured gradually from white (=0) to brown (=15; maximum value in

this group of patients); VAS (visual analogue scale) of disease activity assessed by the patient: coloured gradually from light red (=2) to brown (=7; maximum value in this group of patients) ; nm=not measured.

PCA with the 24 significant proteins in SF separated RA from OA (**C**). PCA with the 17 significant proteins in serum separated RA from both, OA and HD (**D**). TNF, IFNα and IFNγ were non-significant and in the majority of patients below detection limit. However, these proteins were elevated in both, SF and serum, of a few RA patients, who separated from OA and HD, suggesting a subgroup of RA.

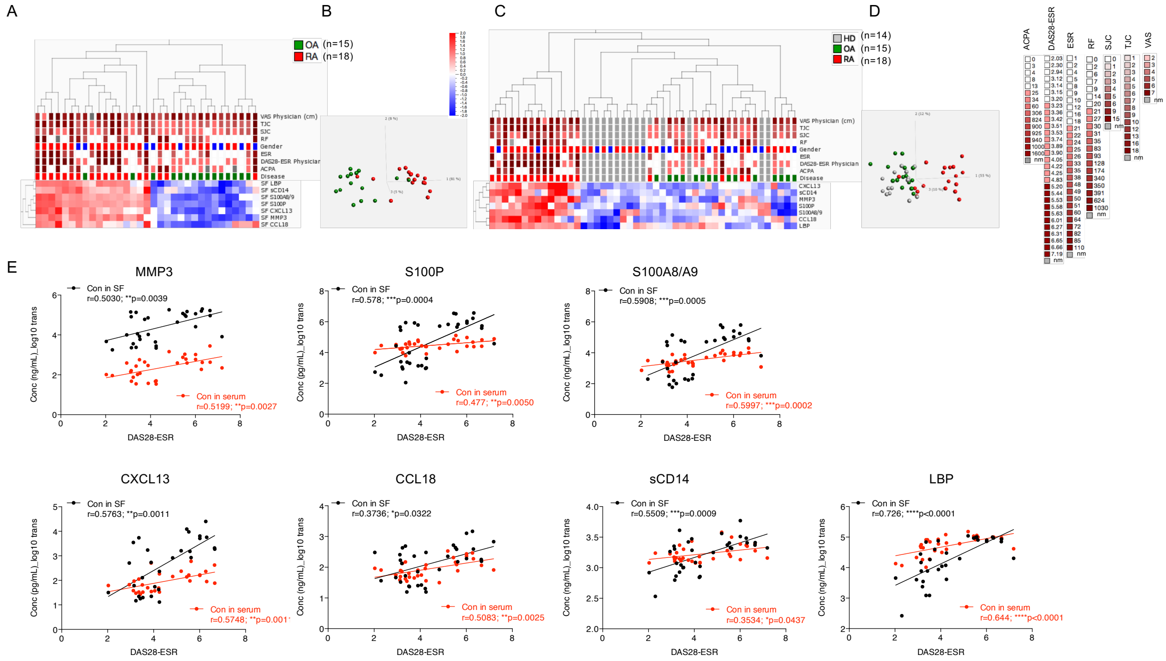

Supplementary figure 11. Protein levels of leading inflammatory molecules in RA. These inflammation related molecules were significantly different between RA-SF (n=18; red) and OA-SF (n=15; green). Protein profiles are presented by hierarchical clustering (**A**) and principal component analysis (**B**). Significant difference was also found between sera from RA (n=18; red) and OA patients (n=15; green) and/or sera from HD (n=14, grey). Serum profiles were presented by hierarchical clustering (**C**) and principal component analysis (**D**).

Dendrograms of clustered samples from SF (**A**) and serum (**C**) are presented with clinical data: ACPA, RF, gender, ESR, TJC, SJC, VAS and DAS28-ESR. These 7 inflammatory molecules reached statistical significance when correlated with the disease activity score DAS28-ESR and CRP (both in SF and sera) (**E**). Correlation coefficient (r) and significance (p) between DAS28-ESR and concentrations of MMP3, S100A8/A9, S100P, CXCL13, CCL18, LBP and sCD14 were determined by Spearman's correlation test.

Black and red circles present relationships between DAS28-ESR and concentrations in SF and serum, respectively.

The scale bar presents relative intensities of the 7 molecules from lowest values (blue) to highest values (red).

Abbreviations and colours within dendrogram in (A) and (B): ACPA (anti-citrullinated protein antibody): ≤20 coloured in white, >20 coloured gradually from light red (=25) to brown (=1600); DAS28-ESR (disease activity score 28-erythrocyte sedimentation rate): ≤3.2

coloured white, >3.2 to ≤5.1 coloured red, and ≥5.1 coloured brown); ESR (erythrocyte sedimentation rate): ≤20 coloured white, >20 coloured gradually from light red (=21) to brown (=110); gender: female-red and male-blue; RF (rheumatoid factor): ≤20 coloured white, >20 coloured gradually light red (=21) to brown (=1030); TJC (tender joint count): coloured gradually from light red (=1) to brown (=18); SJC (swollen joint count): coloured gradually from white (=0) to brown (=15); VAS (visual analogue scale) of disease activity assessed by the patient: coloured gradually from light red (=2) to brown (=7) ; nm=not measured.

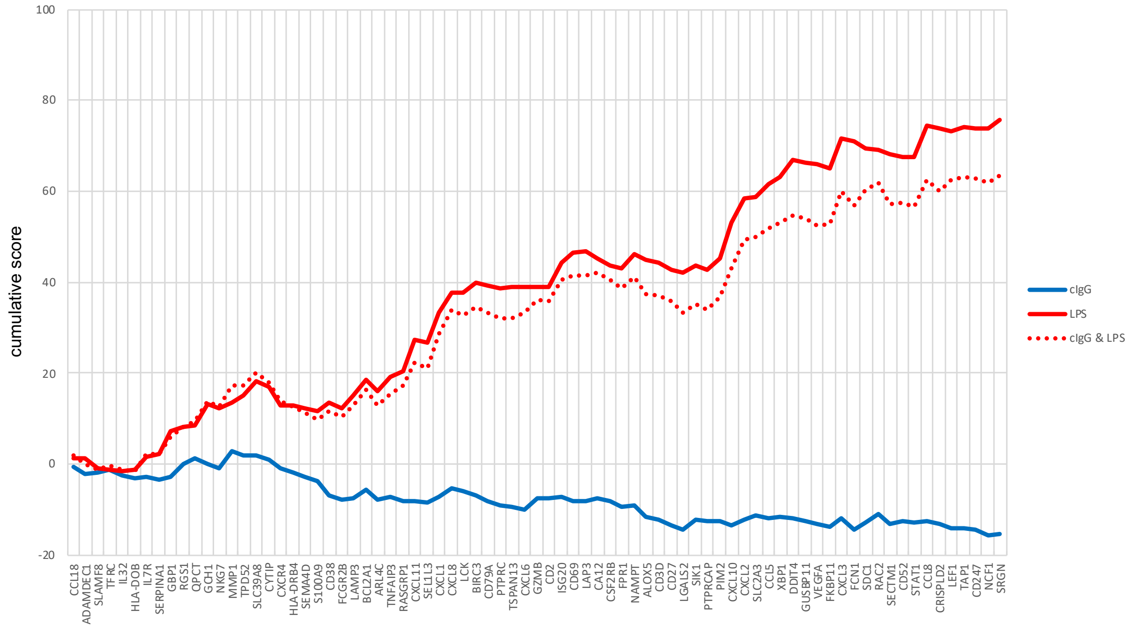

Supplementary figure 12. Quantitative assessment of immunocomplex cIgG (plate coated immunocomplex), LPS and LPS+cIgG activation of monocytes in the top 100 genes encoding secreted/shedded molecules in RA-ST. As described in figure 6 and supplementary figure 7, the top 100 RA-ST genes, which encoding secreted/shedded molecules, were matched with Illumina HiSeq 2000 sequencing data of monocytes unstimulated and stimulated with LPS, cIgG or LPS+cIgG (GSE102728). Overlapping genes (n=77) were identified and are represented by rank on the abscissa according to RA-ST scores. The ordinate shows the cumulative sum of scores, which were determined from the monocyte stimulation experiments with LPS, cIgG and LPS+cIgG by RNA-sequencing. Although determined with a different technology, the cumulative sum of scores for LPS and LPS+cIgG stimulation was comparable to the results with Affymetrix technology. In contrast stimulation with cIgG alone, which mimics deposition of IgG-IC without antigen restrictions, revealed few upregulated genes among top 100 RA-ST genes and an overall negative cumulative scoring. This indicates that cIgG does not sufficiently stimulate monocytes to explain the response pattern, which we observed in RA-ST.

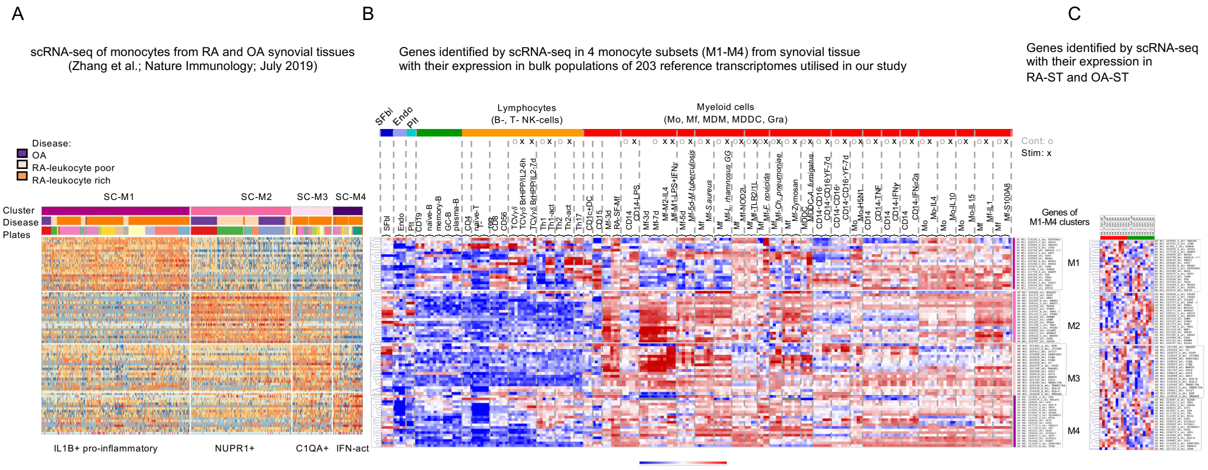

Supplementary figure 13. **Reference transcriptome patterns of genes, which were identified by scRNA-sequencing as characteristic for monocyte subtypes in RA and OA synovial tissues.**

We investigated with our approach the reference transcriptome patterns of genes, which were identified by scRNA-sequencing in synovial tissues and characterized subsets of monocytes (subsets SC-M1 to SC-M4) on the basis of up to 20 genes for each subset (A) (Zhang et al., Nature Immunology July, 2019). The signals of these genes were selected in the 182 reference transcriptomes (**B**), and in our RA-ST and OA-ST transcriptomes (**C**) and hierarchically clustered for each monocyte subset separately. SC-M2 corresponded well to the normal macrophage phenotype with no obvious stimulation patterns. Few genes with patterns of IFN inducibility were found in SC-M4 as suggested by the authors but more were present in SC-M3. Relatedness to bacterial or fungal stimulation patterns were in part found in the SC-M1 but also in SC-M3 and SC-M4. The overall patterning was more heterogeneous and differential expression between RA and OA synovium was less characteristic. Although mostly dominant in monocytes, some of the SC-M1 and M4 genes were also found in T-cells, which were more related to activated Th1, Th2 and $\gamma\delta$-T-cells as well as Th17 cells in the SC-M1 panel of genes. This is comparable with our observations that some genes can be expressed in both cell types, like CXCL13 and TNF. The weaker overlap of SC-M1 and SC-M4 with typical stimulation patterns of microarray reference transcriptomes may relate to the reduced number of detectable genes and the low copy number of differentiating genes in scRNA-sequencing. For example, scRNA differentiating profiles did not reflect many chemokine transcripts, which, however, were observed by the authors when performing bulk sequencing[4]. Transcripts of chemokines and other secreted proteins were even more pronounced in our whole tissue approach and were

an example for the type of genes, which contributed substantially to characterize the different monocyte stimulation patters in our study.

Supplementary table 1. Differentially expressed probe-sets obtained by comparing synovial tissue transcriptomes from RA (n=10) and OA (n=10) patients. In total, 2019 Affymetrix probe-sets were differentially expressed: 1010 were up- and 1009 were down-regulated in RA- compared to OA-ST. Worksheet (WS) 1: 2019 Affymetrix probe-sets with gene names, fold changes (FC) and t-test p-values and scores from comparisons between RA- and OA-ST. WS2: 2019 Affymetrix probe-sets with signal intensities obtained after normalisation as described in section statistical and functional analysis of microarray data.

Supplementary table 2. Top 20 KEGG pathways identified from differentially expressed genes in RA- and OA-ST by gene set enrichment analyses (GSEA). Overview of the leading KEGG pathways with their genes sets, which were presented with enrichment plots in figure 2D-M.

Supplementary table 3. Top 15 biological processes (BP) and cellular components (CC) associated with 2019 Affymetrix probe-sets as determined by DAVID functional annotation tool.

Supplementary table 4. An overview of the reference transcriptomes applied for functional dissection of RA- and OA-synovial tissue (ST) transcriptomes. Initially, 38 reference transcriptomes of 12 different cell types, including the main immune cell types that infiltrate ST, were applied to analyse differentially expressed genes obtained from comparisons between RA- and OA-ST. Further analyses were extended to 182 reference transcriptomes of 64 different data sets, which included the various subsets of the main leukocytes, as well as their differentiation and activation profiles.

Supplementary table 5. An overview of 182 reference transcriptomes and their organisation within heatmaps. Selected reference transcriptomes are organised in such way that control samples are position before corresponding stimulation. In 4 experiments, more than

one stimulus was investigated and compared to the same control samples. To illustrate the differences between stimulation and control, these control samples are shown repeatedly (heatmap in figure 4 and supplementary figure 6). This resulted in the presentation of 203 reference transcriptomes, of which 182 were unique.

Supplementary table 6. Overview of comparisons between reference transcriptomes. In total, 35 comparisons were performed to determine the cell type specific, pathogen triggered and differentiation profiles.

Supplementary table 7. The cumulatively added scores as percentage of the cumulative RA-ST scores. The gene scores determined in RA-ST are ranked from 1-100 as described in supplementary file. Worksheet **WS-A** lists top 100 RA-ST genes in figure 5; **WS-B** lists top 100 genes encoding secreted/shedded molecules in RA-ST in figure 6 and supplementary figure 7**; WS- C** lists top 100 genes in OA-ST showed in supplementary figure 4; **WS-D** lists top 100 genes encoding secreted/shedded molecules in OA-ST in supplementary figure 8. **WS-E** lists the top 100 genes of each of the 35 reference comparisons (RefC) in supplementary figure 5. These 100 genes were listed in column "C" to "AN" together with top 100 RA-ST genes (showed twice in column "B" and "AQ"). The overlap of between top 100 gene of each of the 35 reference comparisons with the top 100 RA-ST genes are included in column "AR" to "CC". The numbers in column "AR" to "CC" represent the rank of the gene in the corresponding reference comparison.

Supplementary table 8. Clinical characteristics of RA and OA patients from whom 1) synovial biopsy samples for transcriptome analyses and 2) synovial fluid and serum samples for protein measurements were collected.

Supplementary table 9. Correlation of soluble markers between synovial fluid and serum, and with clinical data and DAS28. Spearman correlation coefficients and p-values were calculated for the 27 markers 1) between SF and serum protein concentrations in the 33 donors (18 RA and 15 OA) and 2) between clinical data and SF concentrations and 3) between clinical data and serum concentration in the 33 donors.
