# Supplementary material for: Synovial tissue transcriptomes of long-standing rheumatoid arthritis are dominated by activated macrophages that reflect microbial stimulation
Source: Sci Rep. 2020 May 13;10:7907. doi: 10.1038/s41598-020-64431-4 (PMC7220941; doi:10.1038/s41598-020-64431-4)
Supplement: Supplementary file 9 — Supplementary Table 8 [file 41598_2020_64431_MOESM9_ESM.docx]

**Supplementary table 8.**

**Clinical characteristics of RA and OA patients from whom 1) synovial biopsy samples for transcriptome analyses and 2) synovial fluid and serum samples for protein measurements were collected**

**
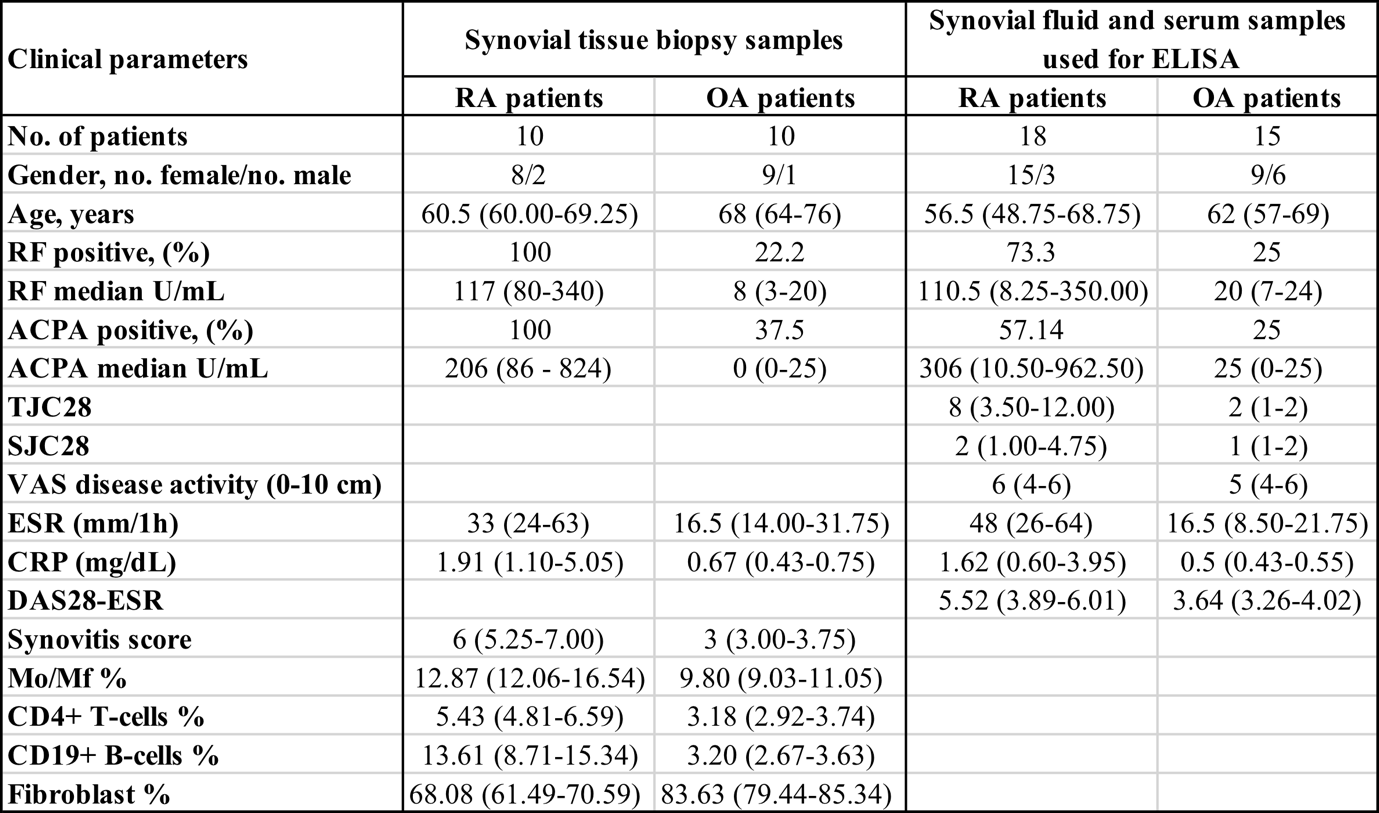
**

Values are median with interquartile range (IQR). RF=rheumatoid factor, ACPA=anti-cyclic citrullinated peptide antibodies, TJC28=28-joint tender joint count, SJC28=28-joint swollen joint count, VAS (cm)=visual analoge scale, ESR=erythrocyte sedimentation rate, CRP=C-reactive protein, DAS28=Disease Activity Score in 28 joints, Synovitis score: 0-1 (no synovitis), 2-4 (low grade synovitis), 5-9 (high-grade synovitis), infiltration of Mo/Mf, CD4+T-cells, CD19+ B-cells and fibroblasts.
